# Spatiotemporal network coding of physiological mossy fiber inputs by the cerebellar granular layer

**DOI:** 10.1371/journal.pcbi.1005754

**Published:** 2017-09-21

**Authors:** Shyam Kumar Sudhakar, Sungho Hong, Ivan Raikov, Rodrigo Publio, Claus Lang, Thomas Close, Daqing Guo, Mario Negrello, Erik De Schutter

**Affiliations:** 1 Computational Neuroscience Unit, Okinawa Institute of Science and Technology, Onna-son, Okinawa, Japan; 2 Laboratory of Theoretical Neurobiology and Neuro-engineering, University of Antwerp, Wilrijk, Belgium; 3 Department of Psychology, University of Michigan, Ann Arbor, Michigan, United States of America; 4 Bernstein Center of Computational Neuroscience Berlin, Berlin, Germany; 5 Department of Neuroscience, Erasmus Medical Center, Rotterdam, the Netherlands; George Mason University, UNITED STATES

## Abstract

The granular layer, which mainly consists of granule and Golgi cells, is the first stage of the cerebellar cortex and processes spatiotemporal information transmitted by mossy fiber inputs with a wide variety of firing patterns. To study its dynamics at multiple time scales in response to inputs approximating real spatiotemporal patterns, we constructed a large-scale 3D network model of the granular layer. Patterned mossy fiber activity induces rhythmic Golgi cell activity that is synchronized by shared parallel fiber input and by gap junctions. This leads to long distance synchrony of Golgi cells along the transverse axis, powerfully regulating granule cell firing by imposing inhibition during a specific time window. The essential network mechanisms, including tunable Golgi cell oscillations, on-beam inhibition and NMDA receptors causing first winner keeps winning of granule cells, illustrate how fundamental properties of the granule layer operate in tandem to produce (1) well timed and spatially bound output, (2) a wide dynamic range of granule cell firing and (3) transient and coherent gating oscillations. These results substantially enrich our understanding of granule cell layer processing, which seems to promote spatial group selection of granule cell activity as a function of timing of mossy fiber input.

## Author summary

The cerebellum is an organ of peculiar geometrical properties, and has been attributed the function of applying spatiotemporal transforms to sensorimotor data since Eccles. In this work we have analyzed the spatiotemporal response properties of the first part of the cerebellar circuit, the granule layer. On the basis of a biophysically plausible and large-scale model of the cerebellum, constrained by a wealth of anatomical data, we study the network dynamics and firing properties of individual cell populations in response to 'realistic' input patterns. We make specific predictions about the spatiotemporal features of granule layer processing regarding the effects of the gap junction coupled network of Golgi cells on a spatially restricted input, in an effect we denominate first-takes-all. Furthermore, we calculate that the granule cell layer has a wide dynamic range, indicating that this is a system that can transmit large variations of input intensities.

## Introduction

The granular layer of the cerebellar cortex consists of populations of granule cells (GrCs), Golgi cells (GoCs), unipolar brush cells, and Lugaro cells [1–3]. GrCs are excitatory [1] and form the largest population of neurons not only in the cerebellum, but also in the entire brain [4]. GoCs are inhibitory and are known as interneurons of the granular layer [1,5]. The granular layer of the cerebellar cortex receives its input from different parts of the brain primarily through mossy fibers [6]. The mossy fibers excite both GrCs and GoCs through their typical axonal boutons called ‘rosettes’ [7,8]. Within the cerebellar cortex, the GrCs excite GoCs through parallel fibers [9–11] and ascending axons [12] and the GoCs in turn inhibit numerous GrCs through sagittal branching of their axons [5,13]. So there exists a feedback loop between the GoCs and GrCs [14], which has a similar structure to that of the pyramidal-interneuron gamma rhythm generation (PING) model of the neocortex [15]. In addition, the GoCs are connected together by gap junctions [16–18] and have also been reported to inhibit each other sparsely [19].

Previous studies have proposed different roles for cerebellar GrCs. Jörntell and colleagues have suggested that GrCs function as signal-to-noise enhancing elements [20,21], based on their observation that GrCs in the C3 zone of decerebrated cats receive identical inputs through mossy fibers that are modality specific, have the same receptive field type and are similarly encoded. Other studies proposed that GrCs provide a bank of various temporal patterns (tapped delay line model, spectrum models) that can be used to generate learned temporal responses such as in classical conditioning experiments [22–25]. According to this view, the GrC population is endowed with a variety of time constants so that the different GrCs are active at different moments during conditioned stimuli [26].

One of the earliest proposals for the GrC function was by David Marr, suggesting that they act as low noise sparse encoders [27], a popular hypothesis supported by some recent electrophysiological and modeling studies [28–30]. In this theory, each GrC represents a combination of a few mossy fibers that provide diverse input, where the number of activated GrCs at any time is small compared to the total number of GrCs, i.e. sparse firing, to facilitate discrimination of binary input patterns by Purkinje cells. In support of this, GrCs receive only a few mossy fiber inputs [31] and exhibit low background firing rates partly due to the presence of tonic GABAergic input [29]. With such a synaptic structure, sparsely firing GrCs could losslessly encode a wide range of spatial input patterns [30].

**Fig 1:**
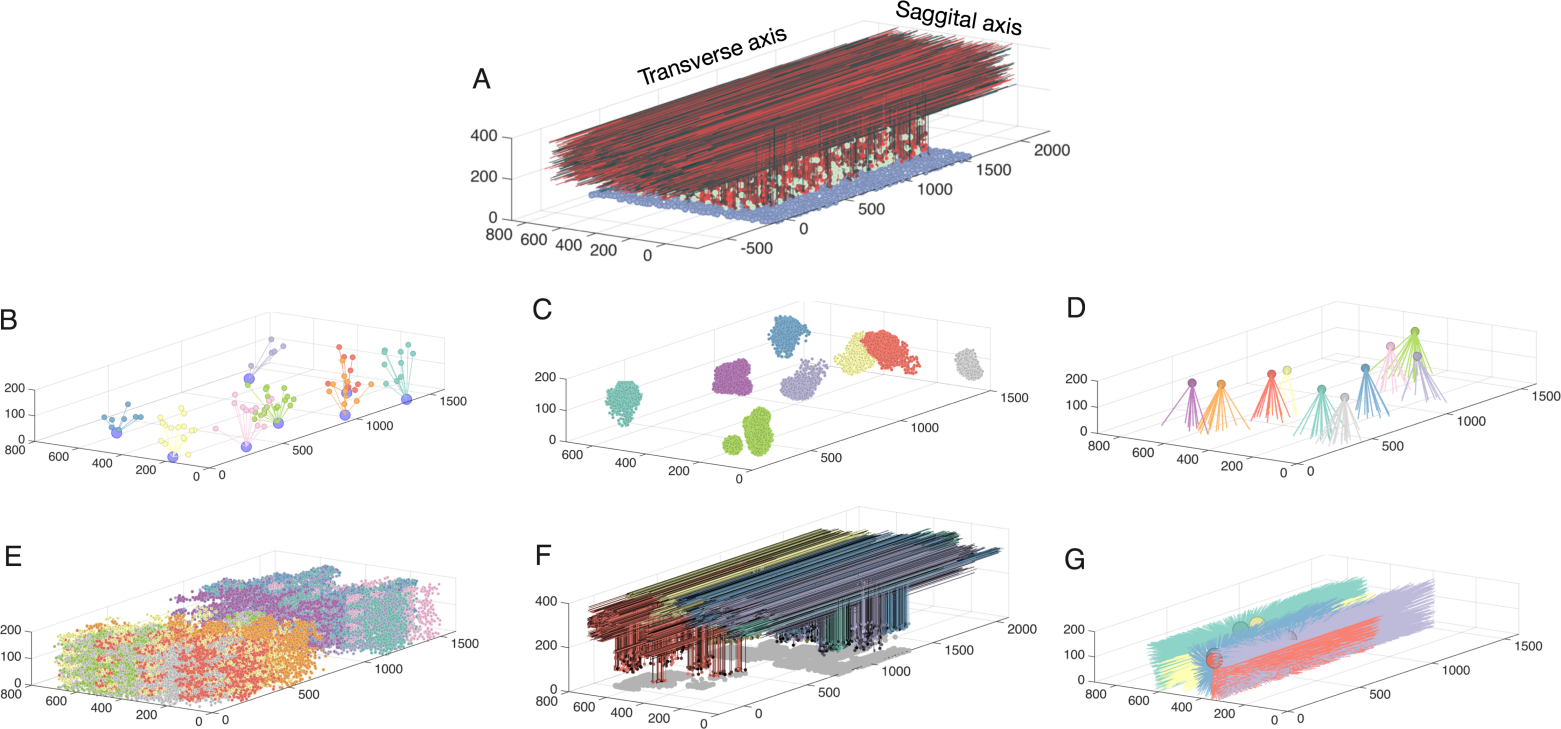
Network connectivity for all populations in the granule layer model. **A:** The completely assembled network showing 10% of all GrCs and parallel fibers (red shades), along with all GoCs (green) and all mossy fibers locations (blue). The following panels always show subsets of model elements to demonstrate their specific connectivity. **B:** Example of 7 mossy fibers diverging onto GoCs. **C:** The GrCs contacted by 8 different mossy fibers through rosettes (color coded by mossy fiber identity). **D:** Mossy fibers converging onto different GoCs (one color per Golgi cell). **E.**: GrCs inhibited by different Golgi cells (each color represents one Golgi cell). **F:** Ascending axons and parallel fibers relating to 5 different mossy fibers (each color represents one mossy fiber) **G:** Parallel fibers from granule cells, which converge onto different GoCs (each color represents one Golgi cell).

Sparse encoding assumes relatively uncorrelated GrC activity, however, there are multiple anatomical and physiological mechanisms that promote correlation of GrCs. For instance, it has been demonstrated that bursting of a single mossy fiber afferent can lead to bursting of many GrCs [32]. Furthermore, granule cells in the flocculus respond with high mutual correlation during vestibule-ocular reflex tasks, due to the activation by unipolar brush cells [33]. In addition, spillover mechanisms could conceivably create spatially correlated GrC input [34]. The sagittal arrangement of mossy fiber rosettes are likely to create anisotropic spatial correlations [7,35]. Recent in vivo imaging of the granular layer reports a lack of sparse activity in GrCs [36]. Hence, a detailed analysis on how granular layer network mechanisms contribute to spatiotemporal encoding of parallel fiber activity is timely, particularly considering the anatomical interactions between the cell populations characterized by the high density of GrCs [4] (Fig 1A–1F).

Early physiologically detailed models of the granular layer, constructed in 1D and 2D, have suggested the presence of robust oscillations in the granular layer of the cerebellar cortex due to the feedback loop between GoC and GrC’s [14,37]. The oscillations cease if there is very low mossy fiber activity, or a dominant excitation of GoCs by mossy fibers or high tonic inhibition of GrCs in the network [14,37,38]. The 2D model suggested that gap junctions between GoCs increase the power of feedback loop driven oscillations [37]. Recently it has been suggested that the emergence of network oscillations can also be linked to NMDA receptors at parallel fiber-GoC synapses [39]. Comparable oscillations have also been experimentally observed in the local field potentials (LFP) recorded in the granular layer, in the 10–25 Hz range in the paramedian lobule of primates [40] and in the range of 7–8 Hz in Crus IIa of awake rats [41].

While previous detailed computational models were studied with a limited repertoire of mossy fiber stimuli such as spatially uniform and monotonic ones, etc. [14,37,39], the mossy fiber firings *in vivo* exhibit a variety of temporal and spatial patterns. Vestibular mossy fibers provide slow rate-coded inputs that linearly encode head velocity [33,42,43]. In response to sensory stimulation, mossy fibers in Crus I and Crus IIa generate high frequency bursts [29,32], and metronome mossy fibers of the lateral reticular nucleus (LRN) spike synchronously [44,45]. Furthermore, in response to peripheral stimulation, each body part is represented multiple times in the form of patches in the granular layer, where adjacent patches represent non-adjacent body parts, forming a so called fractured somatotopy [46,47]. Here we simulate a 3D large-scale network model of the granular layer activated by patches of mossy fibers inputs with realistic firing patterns such as slow rate modulation or rapid bursting, to study how spatiotemporal interactions between the neurons determines holistic network dynamics.

## Results

### Tunable oscillations characterize the network dynamics

First, we simulated the granular layer network (Fig 1) model with spontaneous background firings of all mossy fibers. The mossy fiber firing rate was 5 Hz, which is comparable to experimental observations: Cuneate mossy fibers fire spontaneously around 9 Hz *in vivo* [21]. Mossy fibers of the LRN of the brainstem fire regularly (spontaneous) in a wide range from 2–23 Hz [21,45]. Mossy fiber boutons *in vivo* from crus I and crus II of cerebellar cortex are spontaneously active around 4 Hz [32]. With the background inputs, the GrCs and GoCs in the model fired with a mean frequency of 1.01±0.09 Hz and 8.18±0.61 Hz respectively, and these match values from *in vivo* recordings, ~1 Hz (GrCs in Crus I-IIa anaesthetized of rats) [29] and ~8 Hz (GoCs in Crus I-II of anaesthetized rats) [48].

**Fig 2:**
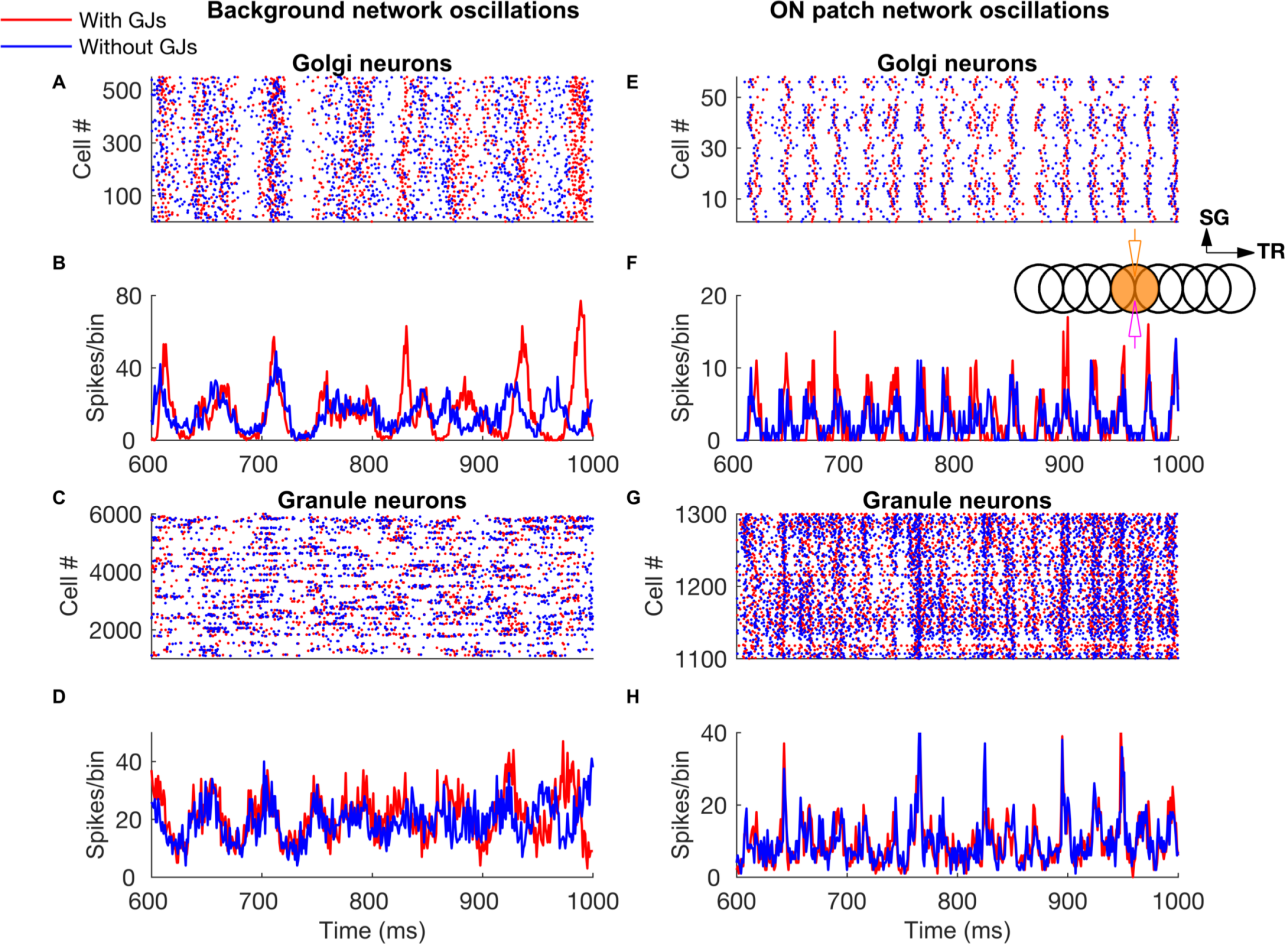
Network-wide oscillations with mossy fiber inputs. **A-D:** GoC and GrC activity in the presence of 5 Hz background mossy fiber input. A and B represent GoC population raster plot and population spike timing histogram, respectively. C and D are the same type of plots as A and B for the GrC population. **E-H:** GoC and GrC activity when mossy fibers within a patch (a red circle in Fig 1) are activated at 60 Hz in addition to the 5 Hz mossy fiber activation of the rest of the network. E, F: GoC raster plot and population spike timing histogram. G, H: the same plots for the GrCs. The inset represents stimulation and recording configuration used for E-H. An orange and empty region represents the stimulated (ON) and unstimulated (OFF) patch, respectively. Electrodes represent stimulation (orange) and recording (magenta), respectively. TR: Transverse. SG: Sagittal.

A characteristic feature of the network activated with diffuse mossy fiber input is widely distributed oscillations of GoCs and GrCs (Fig 2), driven by the feedback loop from GoCs onto GrCs, and vice-versa. The loop consists of AMPA and NMDA receptors of the GrCs activated by the mossy fiber input, AMPAergic receptors in the GoC population activated by the parallel fiber/ascending axon input, and GABAergic receptors in the GrCs activated by the GoCs. Oscillations in baseline could readily be seen in single cell activities of GoCs (Fig 2A and 2E), but were less obvious in single GrC firing for background input, as they are sparsely active (Fig 2C). As observed in a previous 2D network model of the granular layer of cerebellar cortex [37], gap junctions between GoCs increased the synchrony of GoC and of GrC firing in case of low frequency diffuse mossy fiber input but had less effect when in addition a patch of mossy fibers was activated more strongly (compare Fig 2B and 2D).

**Fig 3:**
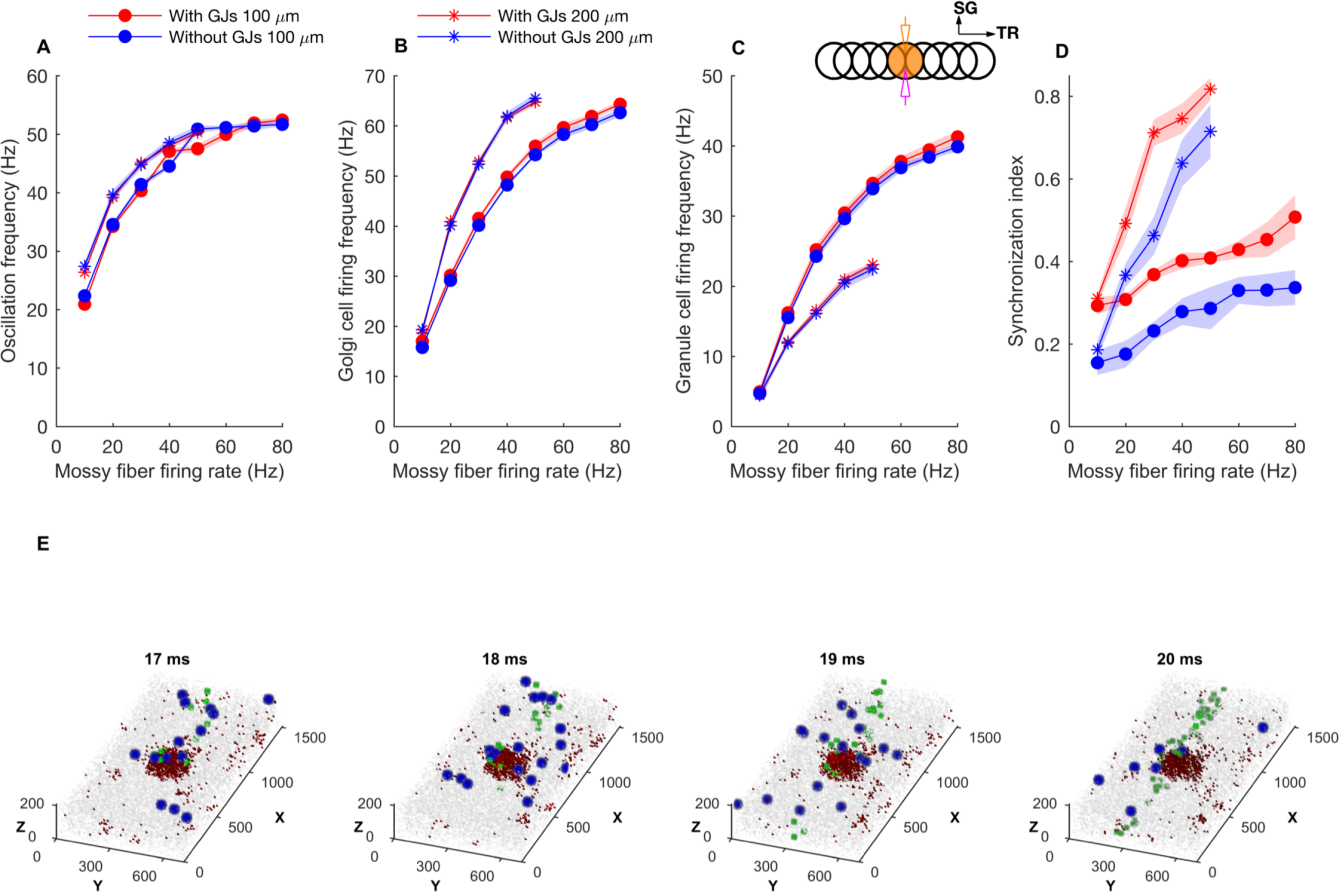
Oscillation frequency and firing rates for patch activation with different input firing frequencies. **A:** network mean oscillation frequency versus average mossy fiber firing rate versus for 100 and 200 μm ON patch radius, with and without gap junctions. **B, C, D:** Same as A for mean GoC firing rate, mean GrC firing rate, network synchronization index, respectively. Membrane potentials of example neurons are shown in S1A–S1D Fig. With the 200 μm patch radius, mossy fiber firing rates above 50 Hz caused depolarization block of GoCs and this data was discarded. **E:** Volumetric maps representing network activity at different times during a 60Hz patch stimulus. Full sequence is available in S1 Movie. The blue and green dots represent mossy fiber and GoC activity respectively, while gray dots represent non-spiking neurons. The small red points are active GrCs. Notice, from left to right, the activations of mossy fibers in the central patch, leading to increased GrC activity in the patch and followed by activation of a beam of GoCs. The inset in C represents the same configuration as the one in Fig 2. Data are mean±standard deviation.

The mossy fiber firing rate was vital in controlling the firing rates of GrCs and GoCs and of the network oscillation frequency. In addition to the baseline input of 5 Hz, we activated the mossy fibers in patches of 100 or 200 μm in radius, which we will call the ON patch, over a range of input frequencies. This protocol simulated the patch-like mossy fiber activations observed *in vivo* [49]. In those simulations, GoCs showed highly synchronized oscillations (Fig 2F) while GrCs exhibited more loose synchronization (Fig 2H). Oscillation frequency increased with the frequency of the activated mossy fibers (Fig 3A) together with the firing frequency of GoCs (Fig 3B) and GrCs (Fig 3C), regardless of the patch size. The presence of gap junctions slightly increased the firing frequency of GoCs (Fig 3B) and GrCs (Fig 3C) for high mossy fiber firing rates. Unlike the previous one-dimensional model of granular layer [14] where oscillations disappeared for mossy fiber firing rate below 15 Hz, oscillations were still observed in our model for 5 Hz mossy fiber background firing rate.

Each model GoC needed to receive inhibition on its apical dendrites to obtain experimentally observed firing rates (see Methods). The origin of this inhibition is not conclusively known, but we postulated that it largely originates from nucleocortical neurons [50,51], with a total conductance for each GoC of 2160 pS based on experimental results. This made GoCs fire in the experimentally observed range *in vivo*. Each GoC also received inhibition on basal dendrites from other GoC cells [19]. This basal inhibition was about ~10 pS/Hz*(average firing rate of presynaptic GoCs), which is much weaker than the inhibition on apical dendrites. However, the effect of GoC-GoC inhibition remained limited even when it was artificially increased. Increasing the synaptic conductance of GoC-GoC inhibition 8 times decreased the GoC firing rate only moderately from ~64 Hz to ~54 Hz with mossy fibers constantly firing at 80 Hz. The same condition changed oscillation frequency barely (from 52.1 Hz to 54 Hz), but the oscillation power decreased to 45%. Therefore, increased GoC-GoC inhibition weakened the oscillations, with small effect on their frequency.

At the single neuron level, the response to mossy fiber input was quite stochastic (Figs 3E and S1. See also S1 Movie). This paper, an initial description of our results, will mostly emphasize the average behavior of the network, which is quite complex. But this only summarizes the rich and stochastic network dynamics, shown in the Supplementary Movies.

The synchronization index of the GoCs and GrCs increased with the firing frequency of mossy fibers in the activated patch and with the size of the patch (Fig 3D), as a larger number of GoCs became involved in each oscillation. Elimination of gap junctions reduced the synchronization index but did not eliminate synchronization at higher mossy fiber firing rates.

### High dynamic range of granule layer encoding

**Fig 4:**
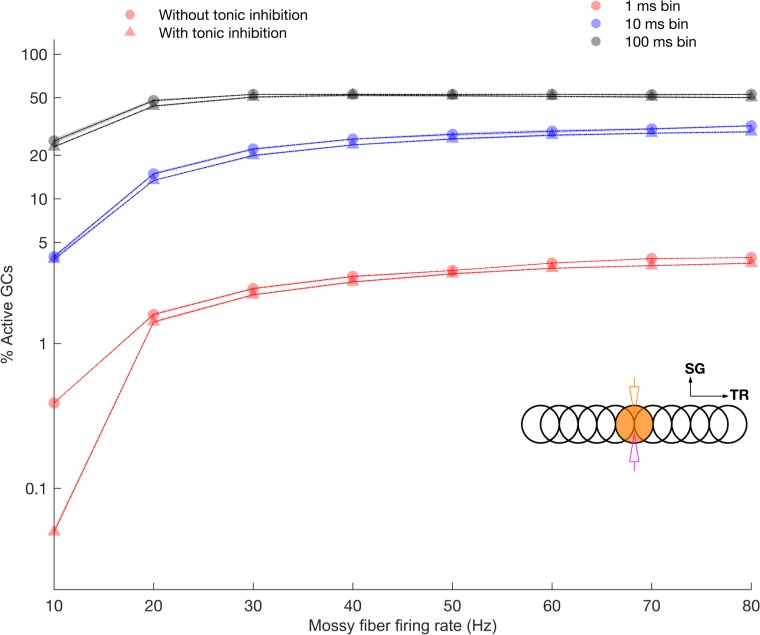
Dynamic range of GrC activation quantified for ON and OFF patches in response to varying mossy fiber firing rate. Percentage of active GrCs computed with different time windows for ON patch in the presence and absence of tonic inhibition.

The divergence rate from a single mossy fiber to GCs is quite large (see S1 Table). Moreover, mossy fiber rosettes occupy a restricted volume of the granular layer allowing for activation of circumscribed patches (Fig 1C). The sparse coding hypothesis predicts that GrC firing should remain sparse, also for high input conditions. However, in our model the GrC activity within the activated volume climbed quickly with increasing mossy fiber input frequency, from just a few cells to about half the cells in a patch (in a long integration window of 100 ms, Fig 4A). We defined the dynamic range as the ratio of maximal to minimal activation of GrCs (see Methods). The largest dynamic range was found for a physiologically relevant integration window of 1 ms, where increasing the mossy fiber firing rate from 10 to 80 Hz caused 10.1 times more GrCs to fire in the ON patch. The sparse baseline firing rate of the GrCs likely represents an almost quiescent network, and this sparse firing quickly transforms into dense firing upon stimulation. Adding tonic inhibition (see the last section of Results) slightly increased the dynamic range.

### Network response to input with slow firing rate modulation

**Fig 5:**
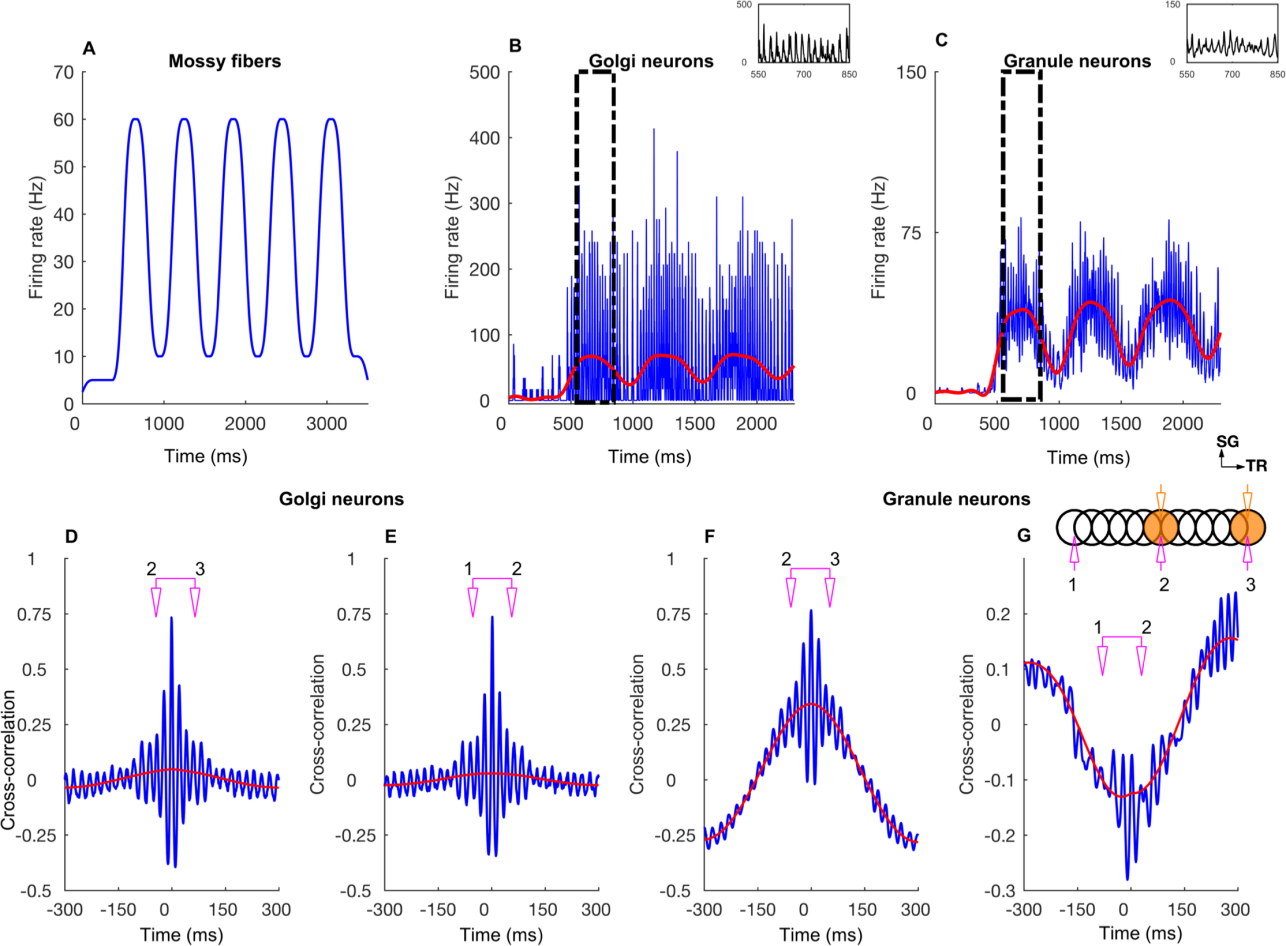
Network response to slow rate modulated mossy fiber inputs activated in two patches along the transverse axis. **A:** Average mossy fiber firing rate in the ON patches, alternating between 10 and 60 Hz. **B:** Average GoC firing rate within the ON patches. The inset is a zoomed view of the boxed region. The blue trace represents the firing rate computed with a 1 ms time bin. The red trace is obtained by low-pass filtering (< 10 Hz) the blue trace. Membrane potentials of example cells are shown in S1E and S1F Fig. **C:** Same as B for GrCs. Membrane potentials of example cells are shown in S1G and S1H Fig. **D:** Cross-correlation function (CCF) between GoC firings in two ON patches separated by 500 μm along the transverse axis. The blue and red curves represent which data the CCF is computed from and follows the same scheme as B and C. The scheme in pink refers to the recording electrode setup shown in the inset of G. **E:** CCF between GoC firings in an ON and OFF patch separated by 500 μm along the transverse axis. Same color conventions. **F, G:** Same as D and E for GrCs, respectively. The stimulation and recording configuration shown as inset in G follows the same scheme as the one in Fig 2.

**Fig 6:**
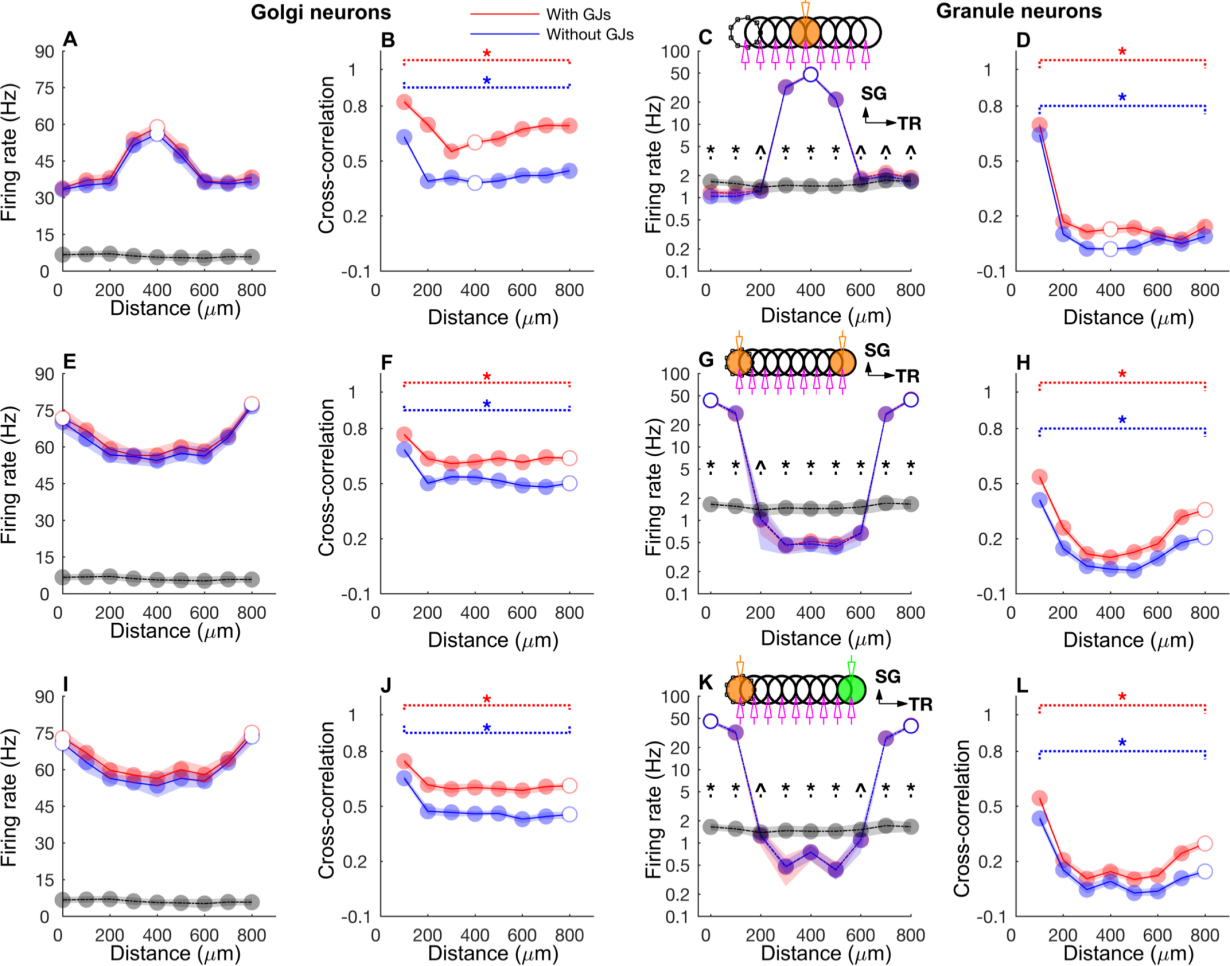
Firing rate and cross-correlations along the transverse axis. **A-D:** GoC firing rate (A), cross-correlation (B), GrC firing rate (C), cross-correlation (D) along the transverse axis when the network was activated with a single patch of mossy fibers with slow rate coded input. Black lines and gray dots represent background network firing rate for the respective patches. Where red (simulations with gap junctions) and blue data (without gap junctions) overlap perfectly, the colors are added resulting in purple. Broken lines in B and C indicate significant correlation throughout the entire range of the data. **E-H:** Same as A-D for the network activated with mossy fibers in two ON patches separated by 800 μm along the transverse axis. The same rate modulation was used for mossy fiber inputs in both patches. **I-L:** Same as E-H, while rate modulation in one of the patches followed the same time course but had different a peak rate of 50 Hz which is marked by green color in the inset in K. Asterisk and triangle represent significant (p<0.01) and insignificant correlation (p> = 0.01), respectively in B, D, F, H, J and L. Similar symbols were used to show the results of two tailed t-tests comparing activity in each patch between patch activation and background activation data in C, G and K. The stimulation and recording configuration shown as inset in C, G, and K follow the same scheme as those in Fig 2. Beaded circle represents the reference patch with which the correlation of every other patch is computed. Data are mean±standard deviation.

**Fig 7:**
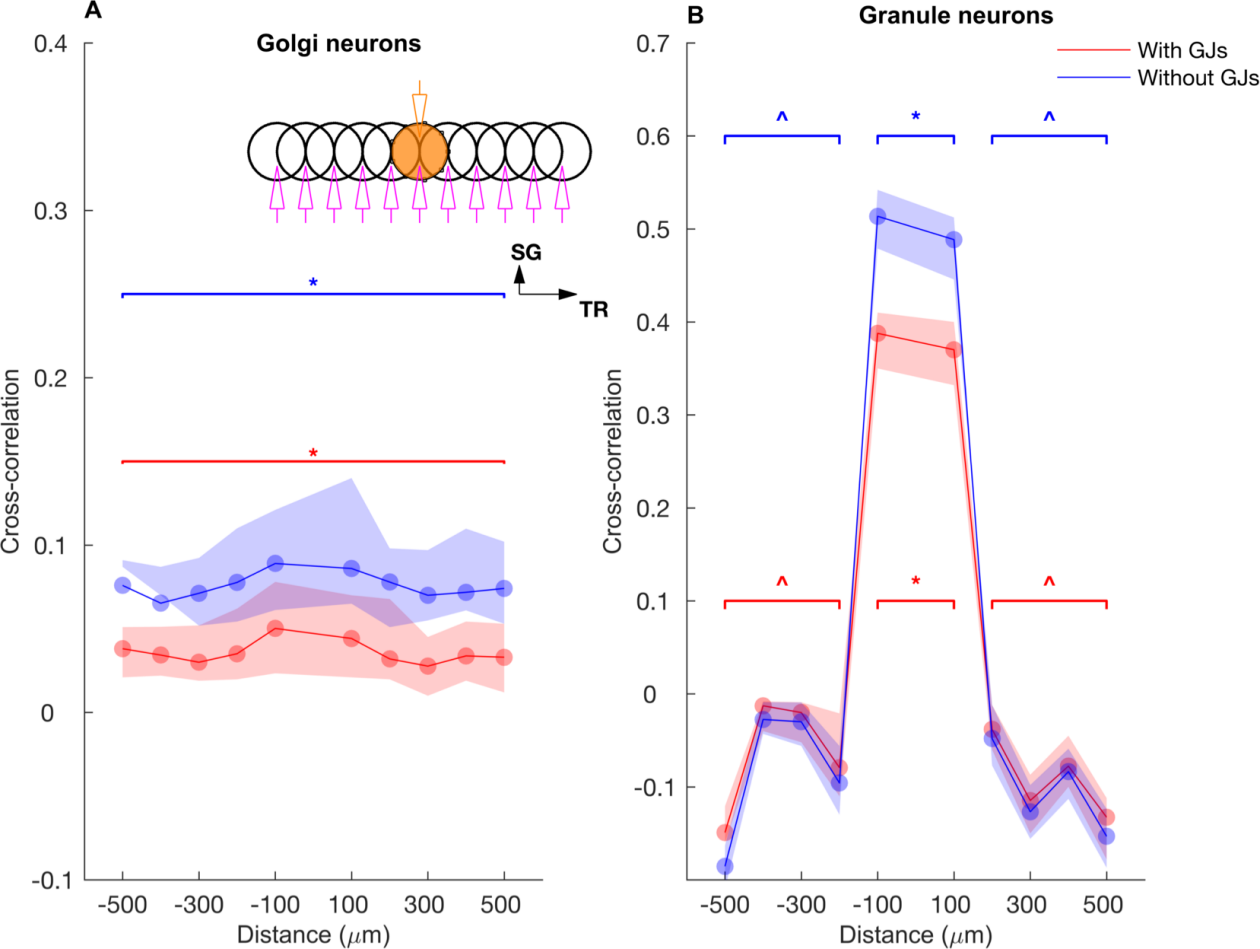
Correlation of slow rate change along the transverse axis. **A, B:** Cross-correlation of long term firing rates for GoCs and GrCs along the transverse axis with the ON patch as a reference (beaded patch). Cross-correlation was computed based on low-pass filtered (< 10 Hz) spike trains. Note that the firing rate correlation is higher in the absence of gap junctions due to a higher amplitude in rate fluctuations with gap junctions (see Methods). Asterisk and triangle represent significant (p<0.01) and insignificant correlation (p> = 0.01), respectively. The stimulation and recording configuration shown as insets in B follows the same scheme as in Fig 6. Data are mean±standard deviation.

Figs 5–7 and S2 Fig demonstrate how the network responded when the input was time dependent, particularly when firing rates were slowly modulated (Fig 5A). For this, we stimulated the mossy fibers in single or double ON patches of 100 μm radius in various configurations that were 1) single patch (Fig 6C, inset) 2) double patches along the transverse axis with 800 μm of the center-to-center distance (Fig 6G and 6K, inset), 3) double patches along the sagittal axis with 400 μm distance (S2H and S2L Fig, inset). Volumetric maps of the network activity in response to a double patch input along the transverse axis are shown in S3 Fig and S2 Movie.

In both the single and double-transverse patch paradigm, GoCs along the parallel fiber axis showed a high degree of oscillatory synchrony with little effect of firing rate co-modulation (Fig 5B). This was observed not only in the cross-correlation between two ON patches (Fig 5D) but also in the cross-correlation of an ON patch with a *non*-stimulated patch, which we will call an OFF patch (Fig 5E). This demonstrated the effectiveness of the common parallel fiber input to the GoCs along the transverse axis. On the other hand, the effect of firing rate modulation was much more pronounced in the population activity of the GrCs (Fig 5C). In particular, the GrCs in the OFF patches (along the transverse direction) showed anti-correlation of their firing rate with the ON patch GrCs, while the synchronized oscillations could still be observed on a shorter time scale (Fig 5G). Therefore, the spatial structure of the average firing rate and the correlations was strikingly different between the GoCs and GrCs. There was only a small spatial dependence of the GoC firing rates along the transverse axis (Fig 6A, 6E and 6I). They exhibited a stable and high cross-correlation along the transverse axis and showed only a slight decay with distance even after discounting the effect of firing rate co-modulation (Fig 6B, 6F and 6J), indicating that the correlation was due to a high degree of synchronization (Fig 5E) driven by the parallel fiber inputs. On the other hand, the GrC firing rates displayed an *on-beam inhibition* [52] featuring an activated ON patch surrounded by laterally inhibited cells along the transverse axis (Fig 6C, 6G and 6K). In a single ON patch paradigm (Fig 6C), the ON patch GrCs fired at 47.7±1.9 Hz while those in the OFF patch (separated by 500 μm) fired at a below-baseline average rate of 0.86±0.11 Hz, due to increased GoC inhibition in OFF patches. The GrCs along the transverse axis were more strongly correlated within and between two ON patches than between the ON-OFF pairs (Fig 6D, 6H and 6L), with a stronger effect of firing rate modulation (Fig 5F). We compared the two-patch condition for identical (Fig 6E–6H) mossy fiber input frequency with simulations where the patches received different input frequencies (Fig 6I–6L), but overall there was little difference suggesting that the spatial input pattern is more important than the input frequencies.

Spatial profiles of cross-correlations of long term firing rates (red curves in Fig 5D–5G) also clearly exhibited these structures for both GoCs (Fig 7A) and GrCs (Fig 7B) along the transverse axis. In the single ON patch stimulation paradigm, the on-beam inhibition is clearly seen in the GrC rate correlations along the transverse axis (Fig 7B). This is a little more pronounced without gap junctions between GoCs since the GrC firing rates becomes less variable due to a decrease of spike synchronization, but gap junctions did not alter the spatial structure qualitatively. Therefore, the synaptic connectivity in the granular layer results in a *winner-take-all* mechanism where OFF patch GrCs get inhibited by GrCs that receive strong mossy fiber input (ON patch) through parallel fiber mediated feedback inhibition.

In the absence of common parallel fiber input, when activity was measured in patches along the sagittal axis in response to slow rate modulation, GoCs were not activated and GrCs were not inhibited in the OFF patches (S2A and S2C Fig). When two patches were activated in this configuration, GrC correlation reduced as the distance between the patches increased (S2H and S2L Fig) and the GoCs exhibited less synchrony (S2F and S2J Fig) than for the transverse configuration (Fig 6F and 6J). Patches of GoCs in this configuration were correlated if they were close to each other (100 μm) but the correlation between the patches rapidly decreased with distance.

As reported previously [37], gap junctions had a limited effect on the synchrony between GoCs along the parallel fiber axis, evoked by patch activation. Without gap junctions between GoCs, the synchrony was reduced among GoCs (for the single patch condition, Fig 6B) along the parallel fiber axis when compared with the control condition but was maintained all along the parallel fiber axis and didn’t exhibit any decay with distance. This was observed even when we activated two mossy fiber patches along the transverse axis with different firing rates (Fig 6J). The difference in correlation between control and gap junction block conditions decreased for two-patch activation due to increased overall parallel fiber activity (Fig 6F and 6J). Removal of gap junctions between GoCs reduced the synchrony of both GrCs (S2D, S2H and S2L Fig) and GoCs (S2B, S2F and S2J Fig) along the sagittal axis.

To summarize, the application of slow rate coded input to the granular layer model of the cerebellar cortex synchronized the GoCs along the transverse axis, resulting in an on-beam inhibition that makes OFF patch GrCs exhibit anti-correlation on a longer time scale and increases the separation between ON patch and OFF patch GrCs with respect to firing rate.

### Ascending axon input desynchronizes GoC activity along the transverse axis

GrC ascending axons, which excite the basolateral dendrites of GoCs [12], play an important role in shaping the activity of the network, especially the GoC synchronization.

**Fig 8:**
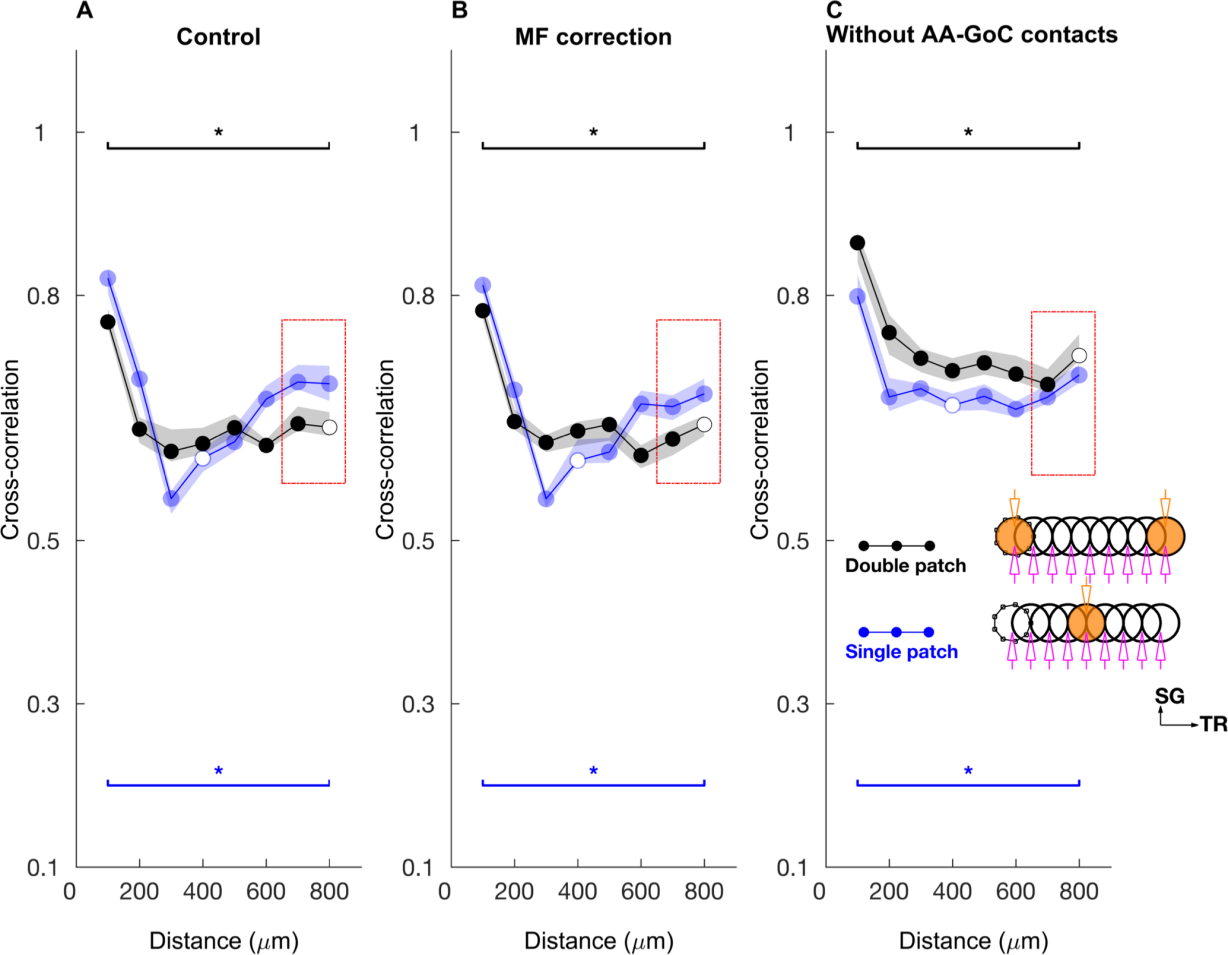
Ascending axon inputs mediate desynchronization of GoCs along the transverse axis. **A:** Cross-correlation of GoCs along the transverse axis for single patch (blue) and double patch activation (black). **B:** Same as A where temporal structure difference of mossy fiber inputs between two ON patches is removed in the double patch activation. **C:** Same as A with ascending axon inputs to GoCs removed. The red box in all the panels indicates the data of interest in the respective curves: the cross-correlation with the remote patch. The stimulation and recording configuration shown as inset in C represents the same configuration as in Fig 6.

In Fig 6 (see also Fig 8A), the cross-correlation of GoCs between two remote ON patches (separated by 800 μm) along the transverse axis (0.64±0.02) is less than that of the corresponding patches (0.69±0.02) for a single patch activation configuration. This is contrary to the intuition that cross-correlation should be larger in the two-patch activation configuration due to an increased activation of shared parallel fiber inputs.

We first checked whether this feature is due to differences in the temporal structure of mossy fiber inputs between the activated patches. We eliminated the difference in temporal input structure between the two activated ON patches (see Methods) and calculated the cross-correlation. This procedure did not affect the cross-correlation in the two-patch activation condition (0.64±0.01; Fig 8B).

Next, we eliminated the ascending axon inputs to GoCs (reduced their peak synaptic conductance to zero) and calculated the cross-correlation in the same manner as above. Fig 8C shows that when ascending axon inputs are blocked, the cross-correlation between the ON patches (0.73±0.03) becomes higher than that of the corresponding patches for single patch activation configuration (0.70±0.02). Moreover, the removal of ascending axon inputs to GoCs, resulted in an overall increase in the cross-correlation for both activation paradigms (Fig 8C).

Because ascending axon inputs represent highly 'localized' input sources to GoCs in both ON patches, they result in reduction of the parallel fiber mediated synchronization. In a single patch activation configuration, cross-correlation varied non-monotonically with distance along the transverse axis and this is due to ascending axon inputs to GoCs (Fig 8A). Here the correlation decreases steeply until 400 μm, which is the last OFF-ON pair, and therefore the ascending axons in the ON patch resulted in a reduced correlation (0.55±0.02). Beyond this point, the correlation is a measure between OFF-OFF pairs and recovers up to 0.69±0.02 at a distance of 800 μm. For the two ON patch configuration, cross-correlation reaches a plateau for distances beyond 400 μm due to the localized ascending axon inputs in the second ON patch (Fig 8A). Increasing the strength of the ascending axon to GoC connections, by increasing their synaptic weights by 20% or 40% of their original values led to a small decrease in cross-correlation of 2–5% along the transverse axis (not shown). We conclude that ascending axon inputs to GoCs reduce the parallel fiber mediated synchronization of GoCs.

### Network response to bursting mossy fiber input

Besides the slow rate modulation that we have used so far, some mossy fibers can also respond to sensory stimulation *in vivo* on a much shorter time scale by bursting at an extremely high frequency of a few hundred hertz [32], sustained for a few tens of milliseconds. This signal is reliably transmitted to the GrCs that show similar bursting [29].

**Fig 9:**
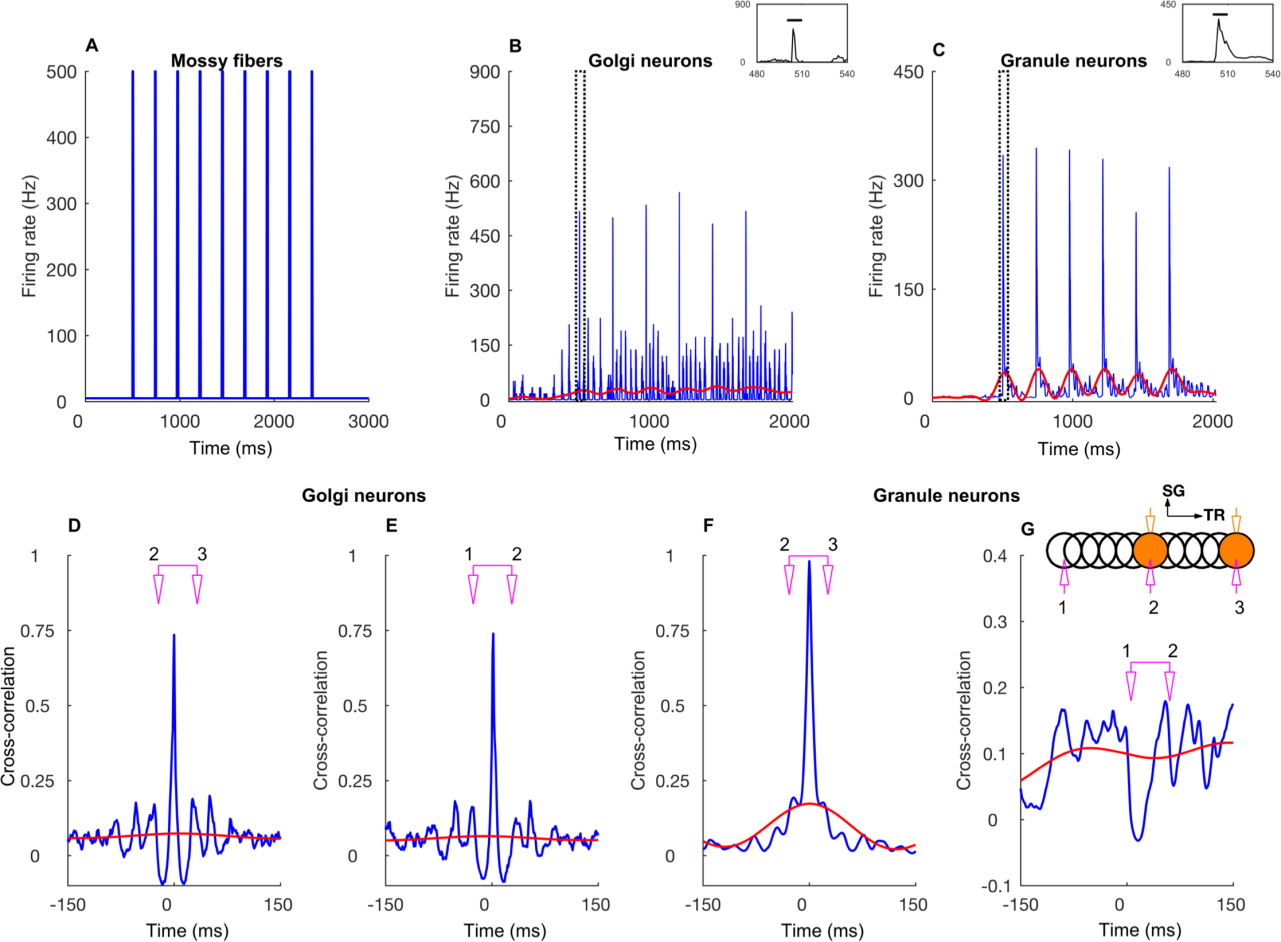
Network response to bursting mossy fiber inputs in two patches along the transverse axis. **A:** Average mossy fiber firing rate in the ON patch. **B:** Average GoC firing rate within the ON patch. The inset is a zoomed view of the boxed region. The blue trace represents the firing rate computed with a 1 ms time bin. The red trace is obtained by low-pass filtering (< 10 Hz) of the blue trace. Membrane potentials of example cells are shown in S1I and S1J Fig. **C:** Same as B for GrC. Membrane potentials of example cells are shown in S1K and S1L Fig. **D:** CCF between GoC firings in two ON patches separated by 500 μm along the transverse axis. The color represents which data the CCF is computed from and follows the same scheme as B and C. **E:** CCF between GoC firings in an ON and OFF patch separated by 500 μm along the transverse axis. **F, G:** Same as D and E for GrCs, respectively. The stimulation and recording configuration shown as inset in K represents the same configuration as the one in Fig 2.

**Fig 10:**
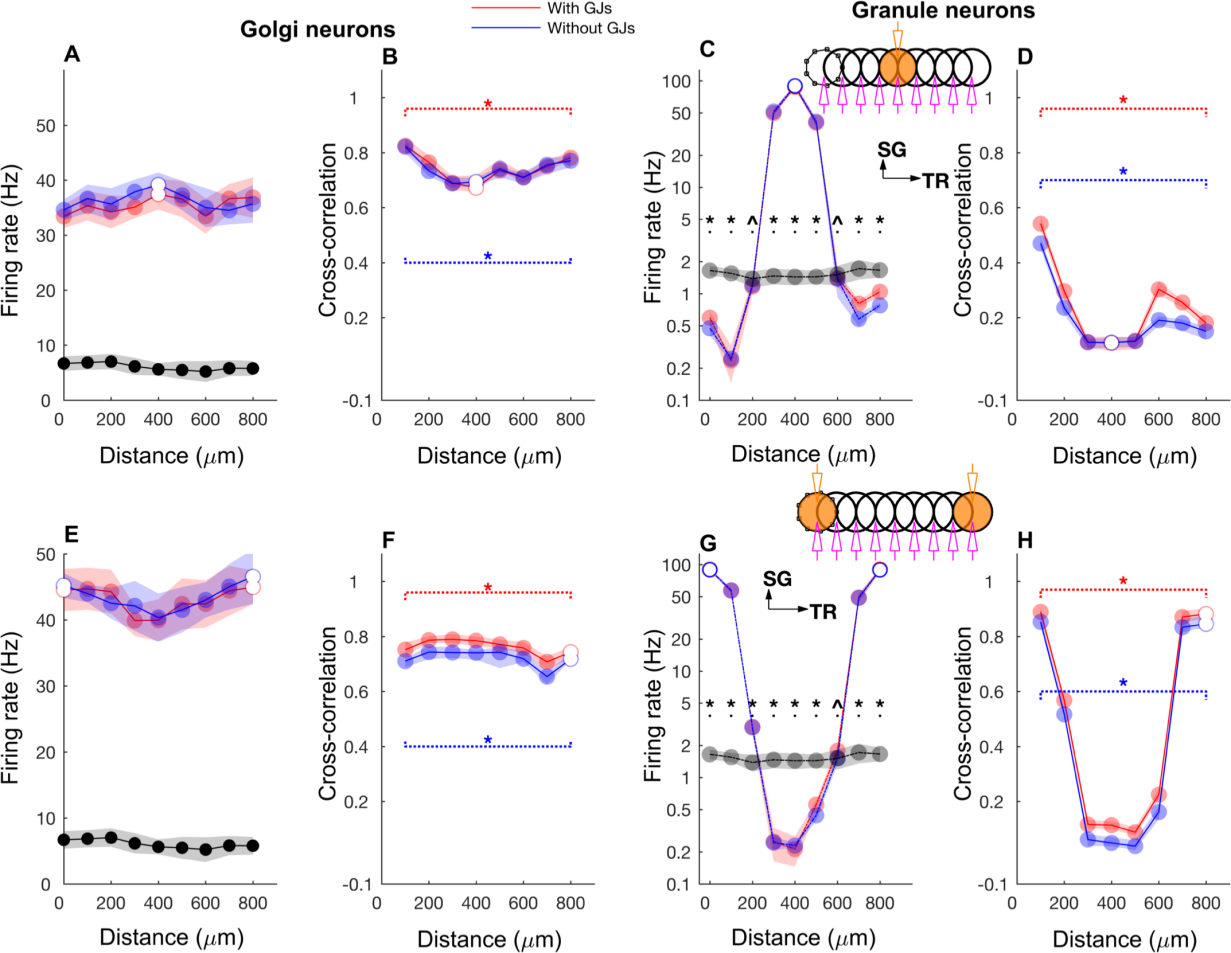
Firing rate and cross-correlation along the transverse axis with bursting mossy fiber inputs. **A-D:** GoC firing rate (A), cross-correlation (B), GrC firing rate (C), cross-correlation (D) along the transverse axis when the network was activated with a single patch of bursting mossy fiber inputs. **E-H:** Same as A-D for the network activated with bursting mossy fiber input in two ON patches separated by 800 μm along the transverse axis. Same color and symbol conventions as in Fig 6.

We simulated this by activating the mossy fibers in the selected ON patch(es) with burst type inputs. Each mossy fiber in the patch was given nine input bursts, with a duration of 10 ms and a firing rate of 500 Hz (Fig 9A). During the 10 ms burst, all the GoCs that fired emitted only one spike (S1I and S1J Fig) and the GrCs spiked 3–4 times (S1K and S1L Fig). As a result, the GoC PSTH showed a sharp peak (Fig 9B, inset), while the GrC PSTH exhibited a prominent broad peak (Fig 9C inset). GrCs were strongly correlated between the activated patches (Fig 9F). GoC firings were highly synchronized for both ON-ON (Fig 9D) and ON-OFF (Fig 9E) patch configurations. All along the transverse axis, GoC firing was sharply synchronized for both single and double patch conditions (Fig 10B and 10F). The ON patch GrCs along the transverse axis were strongly correlated (Fig 10H). The correlation decreased between the ON and OFF patch GrCs and as a result the correlation vs. distance relationship shows the shape of an inverted bell. Volumetric maps of the network activity in response to a single burst activation of a single patch are shown in S4 Fig and S3 Movie.

Along the sagittal axis, the correlation decreased with distance for both GoCs (S5B and S5F Fig) and GrCs, (S5D and S5H Fig) except when the correlation was measured between the two ON patches (S5F and S5H Fig), which showed strong stimulus driven correlations even with a distance of 400 μm between the two patches.

### GoC firing powerfully regulates GrC activity during mossy fiber burst

We observed that the on-beam inhibition of GrCs also emerged with the bursting input (Figs 10C and volumetric representation of cell activities in S4C and S4D) since OFF patch GrCs were silenced due to inhibition by strongly firing GoCs that were activated by the parallel fibers. This on-beam inhibition predicts that GrC spikes can be gated by synchronous GoC firing along the transverse axis. We examined how strongly synchronized inhibition can regulate GrC firing if multiple sets of the mossy fibers along the transverse axis are bursting, particularly when there are relative time delays between the bursts. This replicates *in vivo* conditions, where the patches of mossy fibers could get activated along the transverse axis at various latencies in response to peripheral activation.

**Fig 11:**
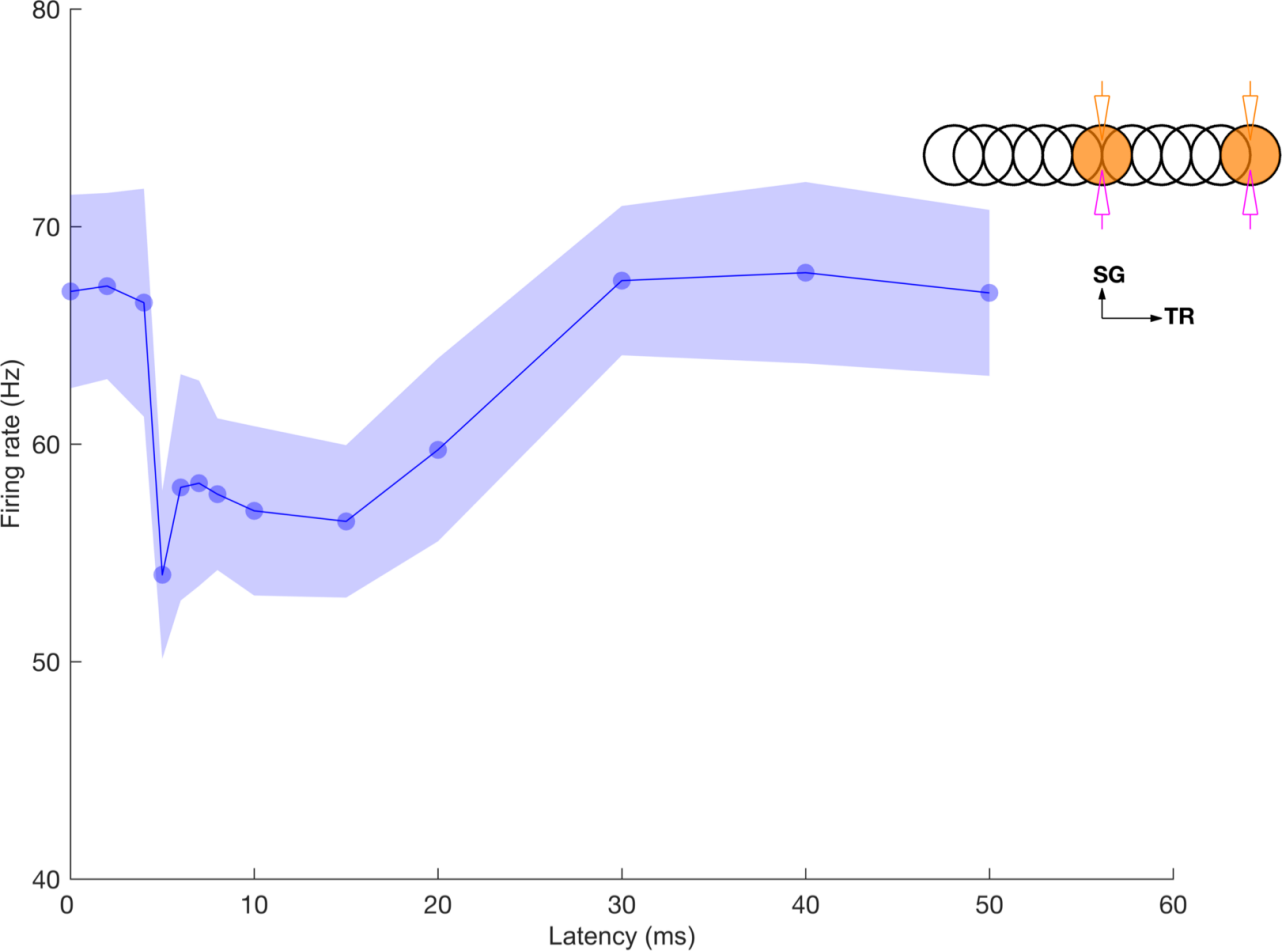
Feedback inhibition reduces delayed bursting of GrCs. Average GrC firing rate within the patch versus latency of the second ON patch mossy fiber burst. Two patches of mossy fibers were activated along the transverse axis and GrCs were recorded in the patch with delayed mossy fiber activation. The stimulation and recording configuration shown as inset follows the same scheme as in Fig 2.

We activated two mossy fiber patches separated by 500 μm along the parallel fiber axis (Fig 11, inset) with the same double patch burst activation paradigm but with different latencies between them. We discovered that the feedback inhibition due to the first patch parallel fibers inhibited the GrC excitation in the second patch when the arrival of the feedback inhibition coincided with their mossy fiber excitation of GrCs. Therefore, when the latency of mossy fibers in the second patch was around 5 ms, the GrC excitation was less effective and GrC firing rate decreased by 20% (Fig 11) compared to synchronous activation of the patches. Because of the slow spillover component of GoC inhibition this effect persisted for intervals up to 15 ms and then slowly declined with a return to baseline firing responses at 30 ms intervals. We conclude that synchronized GoC inhibition can strongly regulate GrC firing, even in the presence of excitatory drive, and this mechanism makes the earliest firing GrCs dominate the network activity. Therefore, it may be more appropriate to describe the competition among inputs in the granular as *first-take-all* instead of *winner-take-all*.

### NMDA receptors mediate *winner-keep-winning* mechanism

**Fig 12:**
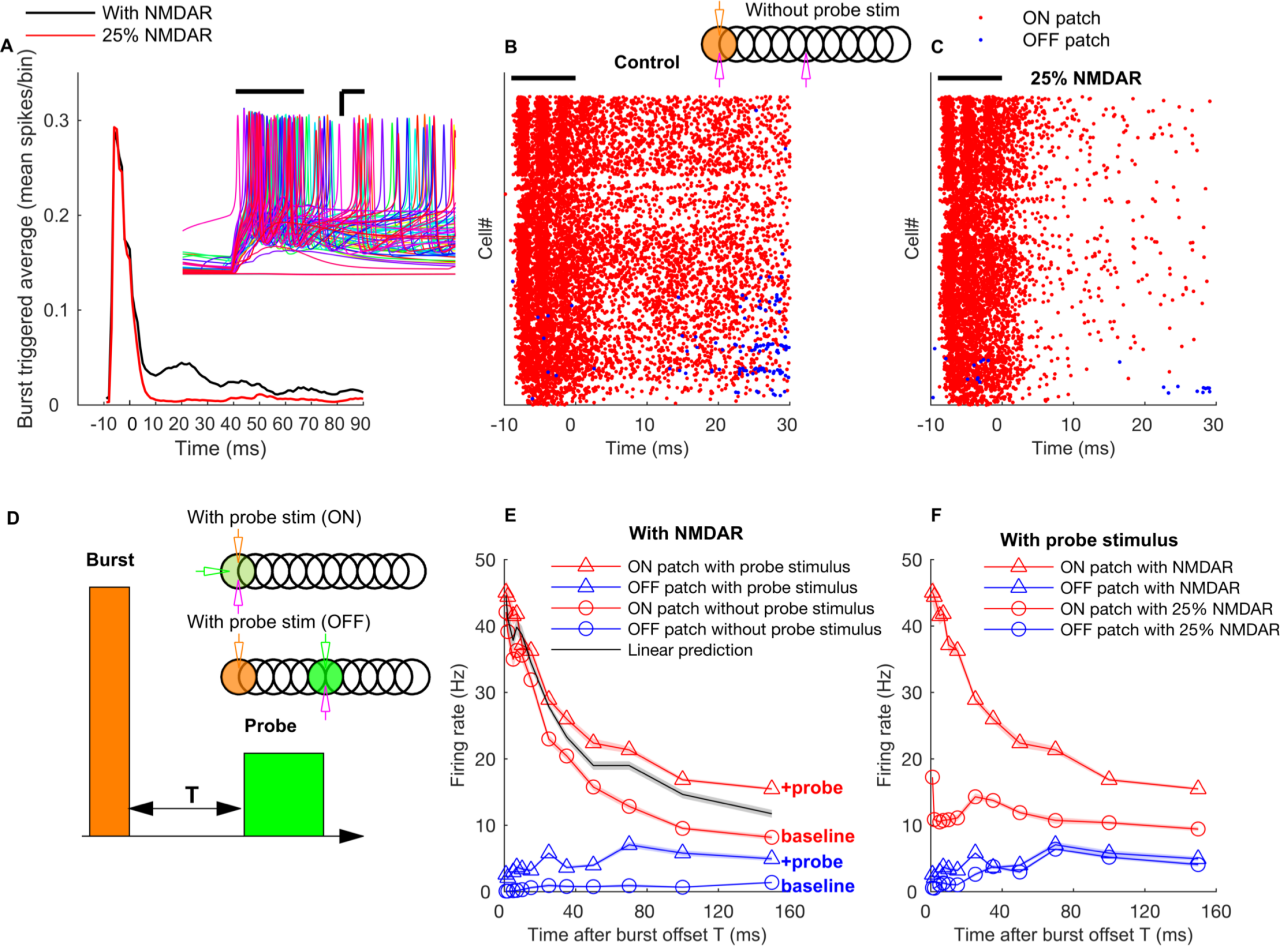
NMDA receptors mediate long-term gain increase in ON patch GrCs. **A:** Burst triggered average firing rate of the ON patch GrCs with normal or reduced conductance of NMDA receptors. Results shown in the absence of probe input. Time zero corresponds to the *end* of the mossy fiber burst. Inset represents membrane potential traces of representative ON patch GrCs in response to bursting mossy fiber input. Scale bar: x-axis = 3 ms, y-axis = 15 mV. **B:** Raster plot of firing of GrCs in ON (red) and OFF patch (blue) in the absence of probe input. **C:** Same as B, with conductance of NMDA receptors reduced by 75%. **D:** Schematic representations of the burst input and probe input. The probe input is presented ‘T’ ms after the burst offset. **E:** Mean firing rate of ON patch and OFF patch GrC population during the asynchronous probe input versus time after the burst offset. Firing rates without a probe input and a prediction assuming no gain change are also shown for comparison. **F:** Mean firing rate of ON patch and OFF patch GrC population with normal and with reduced NMDA conductance during the probe input versus probe input onset time after the burst offset. In A, B, and C, solid line represent burst duration. The inset in B and D represents the stimulation and recording configurations in the same way as in Fig 9 while green color here represents the probe input.

The activity of ON patch GrCs in response to the burst input was not only characterized by firing during the input, but also showed a long transient even after the offset of the mossy fiber burst (black line in Fig 12A). This was unexpected since there are no other sources to excite GrCs other than mossy fibers in our model. We found that the long transient was due to a long-term gain increase in the firing rate by activation of NMDA receptors.

GrCs express NMDA receptors [53] that contain the GluN2C subunit [54] and NMDA mediated currents are known for their non-linear voltage dependence and slow kinetics [55]. The NMDA receptors exhibit voltage dependent block at hyperpolarized membrane potentials due to partial block by magnesium ions, and the membrane needs to be depolarized enough to remove this block. Supralinear synaptic summation [56] can be a mechanism to deliver such depolarization to the NMDA receptors. Therefore, the spiking of GrCs caused by strong mossy fiber input could result in NMDA receptor unblocking and their slow decay kinetics caused effective elevation of the resting membrane potential for a considerable period of time (Fig 12A, inset). In this way, the NMDA receptors can implement a *winner-keep-winning* mechanism, enabling the GrCs that have already spiked to fire again more easily by improved integration of the subsequent inputs.

To further investigate how the effectiveness of this gain change, we delivered a weak probe asynchronous mossy fiber inputs (rate: 20 Hz, duration: 20 ms) with different time delays after the burst offset (Fig 12D). Then, we measured GrC firing during the probe inputs and compared it the control conditions where there is no probe input or when the probe inputs were delivered to an OFF patch. If there is no ON patch specific gain change, the firing rate change in the ON patch GrCs with a probe input should be equal to the rate increase in the OFF patch GrCs due to a probe input. Instead, we found a supra-linear increase in GrC firing, even up to 150 ms after a burst offset (Fig 12E). Therefore, the mossy fiber burst input induced a long lasting gain increase in ON patch GrCs.

The same simulation was repeated with a reduced conductance of NMDA receptors at the mossy fiber-GrC synapse (Fig 12A and 12C). In this case, while there was little change in the response of the ON patch GrC population during the burst, the rebound activity was significantly reduced (Fig 12A and 12C). In response to the probe mossy fiber input, ON patch GrCs exhibited reduced firing rate (Fig 12F) with reduced conductance of NMDA receptors (decrease in firing rate from 44.4 Hz to 10.8 Hz in response to an asynchronous input at 2 ms after the burst offset). The ON patch response amplitudes to the probe stimulus were now closer to those of the OFF patch GrCs. The winner-keep-winning mechanism was robust to changes in the strength of GoC to GrC synaptic inhibition (not shown).

Our results show that the NMDA receptors in GrCs cause a long-term change in their input/output function, which implements a winner-keep-winning mechanism in bursting GrCs. GrCs reliably burst with a single bursting mossy fiber and this has been proposed as a mechanism for reliable signal transmission [32]. Here our results demonstrate that mossy fiber bursts can also be a mechanism for regulating the signal processing property of GrCs and changing how the GrC population filters subsequent mossy fiber inputs.

### Effect of GrC tonic inhibition on network oscillations

GrCs possess extra-synaptic GABAA receptors (receptors with δ subunit) [57,58]. In many neurons of the central nervous system, extra-synaptic GABA receptors mediate a form of 'tonic' GABAergic current and play an important role in their baseline excitability [57].

**Fig 13:**
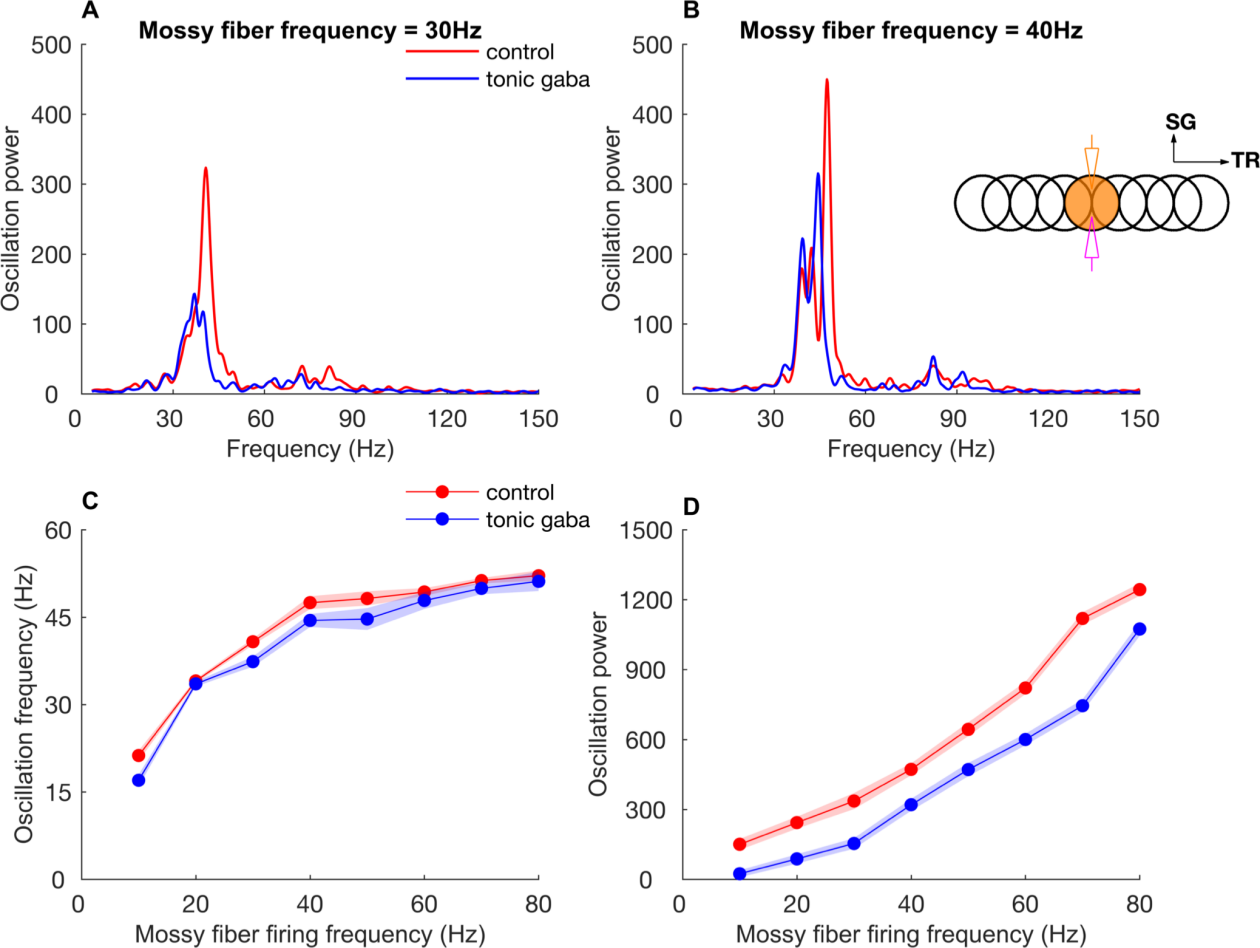
Effect of GrC tonic inhibition on the power and frequency of network oscillations. **A:** Power spectral density of oscillations in firings of ON patch GoCs upon 30 Hz mossy fiber inputs with and without tonic inhibition. **B:** Same as A for 40 Hz mossy fiber inputs. **C:** Oscillation frequency versus ON patch mossy fiber firing rate with and without tonic inhibition. **D:** Peak oscillation power versus ON patch mossy fiber firing rate with and without tonic inhibition. The inset in B represents the same stimulation and recording configuration as in Fig 2. Data are mean±standard deviation.

We simulated the effect of tonic inhibition on network oscillations by quantifying the power and frequency of ON patch network oscillations when activated with constant mossy fiber input of different firing frequencies. We modeled the tonic inhibition as a tonic Cl^-^ conductance of 88 pS in the GrCs (see Methods). We found that the tonic inhibition reduced the power of network oscillations (Fig 13A, 13B and 13D). For a mossy fiber frequency of 30 Hz, tonic inhibition reduced the peak power of GoC network oscillations from 336±34 to 154±22 (Fig 13A). We observed a fairly constant reduction in power of network oscillations for all values of input mossy fiber firing rate (Fig 13D). In contrast, the effect of tonic inhibition on oscillation frequency was small (Fig 13C). For a mossy fiber firing frequency of 30 Hz, oscillation frequency for control condition was 40.4±0.5 Hz while that in the presence of tonic inhibition was 38.2±0.8 Hz (Fig 13C). Therefore, although tonic inhibition of cerebellar GrCs reduces the power of network oscillations, robust oscillation still arise with sufficiently strong mossy fiber input without any effect on the oscillation frequency.

## Discussion

The neural network of the cerebellar granular layer is known as an anatomically well-studied circuitry that only contains a few neuron types, but how it transforms mossy fiber inputs is still being actively debated. Challenges in investigating this question with computational modeling come from the strikingly large imbalance between the number of excitatory and inhibitory neurons and the peculiar geometric properties governing synaptic connectivity. To address these problems, we constructed a large scale and physiologically realistic model of the granular layer neural network that emulates tissue properties of the granular layer of the cerebellum. Rather than introducing assumptions about cerebellar function, our bottom-up approach investigates the dynamical patterns emerging from the known neurocircuitry and physiology.

Another strain of cerebellar models, commonly denominated top-down approaches, aims at capturing functional dynamics that enable motor control [22,23,25,59]. Though some more recent models also include some biophysical and anatomical details, their function relies primarily on the ability of the climbing fiber to modify strengths of parallel fiber synapses to train Purkinje neurons for perceptron-like pattern classification. It is worth noting that other models of sensorimotor control attribute the same abilities to the neocortex [60]. In many models, details are introduced to address limitations of function, such as correcting for saturating activation and synaptic plasticity. For instance, GoCs [27,28,30] and molecular layer interneurons (MLIs) [61] have often been assumed to act like level setting systems or GrCs as tapped delay lines [22–25], a role that may or may not be compatible with their known physiology as described in the Introduction. Such models add biological details to improve the predefined performance of the model.

Our analysis instead focused on spatiotemporal dynamics in response to physiologically plausible mossy inputs and investigated which output patterns emerge in our physiologically detailed model. Through analysis of input/output relationships we showed that synaptic and cellular mechanisms in the cerebellar granular layer enable the network to stochastically transform and integrate information over multiple spatial and temporal scales of mossy fiber input.

### 3D network model of the granular layer

Our model can be considered a superset that reprises findings of previous 1D [14], 2D [36,38] and 3D [38] network models such as feedback oscillations, while suggesting new dynamical phenomena implied by physiology and anatomy. Previous models were significantly smaller and did not analyze network responses as a function of complex mossy fiber activation. Earlier models in [14] and [37] lacked the fine spatial and temporal structure of mossy fiber activation in the cerebellar cortex and did not include the ascending axon input to GoCs. Similarly, the model in [39] approximated mossy fiber input by current injection. A 3D network model in [38] described network dynamics of the granular layer in response to spontaneous and burst input patterns and replicated the center-surround inhibition observed in experiments in sagittal slices, where the parallel fibers are cut [62]. Because of the absence of any significant parallel fiber contribution, this model could not produce the spatial interactions we described here.

Our network model is based on recent conductance based models of individual neuron types [38] and, network topology including both the long folium axis and parasagittal axis in the cerebellar cortex. A potential limitation of our network model is that it extends only for 700 μm along the sagittal axis and therefore does not capture rostro-caudal distribution of a number of structures (e.g., rostro-caudal extent of mossy fiber axonal arborization is usually greater than 1000 μm in the granular layer [7]). The model does not include NMDA receptors at parallel fiber-GoC synapse because they caused depolarization block in the GoC model and have been reported to be absent in adult animals [11,12]. A previous 2D model suggested that NMDA receptors at parallel fiber-GoC synapse can cause activity dependent state transitions in the granular layer [39]. Moreover, the GoC model used in our study does not incorporate the fine branched morphology of GoC dendrites found in the granular layer [11]. Gap junctions between GoCs probably occur more frequently along the sagittal plane of the folia as GoC dendrites follow the zebrin boundaries of Purkinje cells above them [63]. But lack of proper experimental data forced us to model them without any directional dependence. Finally, to achieve physiological low GoC firing rates it was necessary to include dendritic inhibition from sources outside of the granular layer. Inhibition of GoCs has been an unresolved issue since a recent study claimed that MLIs do not inhibit GoCs [19,51] and our results emphasize the importance of extracortical inhibition for normal GoC function. Conversely, the effect of GoC to GoC inhibition is modest due to its weak strength [19].

### Oscillations in the granular layer

One of the strongest features of the simulated network dynamics is the network wide oscillation driven by a feedback loop between the GrCs and GoCs, and mediated by the parallel fibers, which synchronize the GoCs. The synchronized activity of GoCs, were stronger all along the transverse axis as in experimental studies [10]. Consistent with this result [10], we found that common parallel fiber input drives the synchronized spiking in GoCs, but the gap junctions also contributed significantly [18,64] particularly when mossy fiber firing frequency was low. The GrCs in the network, whose spike timings are controlled by cycles of GoC inhibition, exhibit a less precise synchronization.

Afferent mossy fibers that project to the granular layer exhibit a wide variety of firing patterns. Experimental studies have shown that mossy fibers exhibit slow rate modulation during a variety of behaviors [42,65], but can also exhibit burst activity in response to sensory stimulation [20,21,66] firing at more than 700 Hz [32]. We studied how the network responds to and encodes these different physiological input patterns. With one exception, the observed synchronization patterns differed little. This may seem surprising because the mechanisms are fundamentally different, driven by feedback inhibition for the slow rate modulated input, and caused by locking to the strong stimulus for burst input. In response to both types of input, GoCs exhibited parallel fiber mediated synchronization extensively along the transverse axis and this GoC activity powerfully regulated GrC firing. As a consequence, the GrCs correlations showed much more dependence on their location relative to the stimulus than on stimulus type or frequency. Separate GrC populations along the transverse (parallel fiber) axis, fired with significant correlations only when they both received mossy fiber inputs, regardless of whether the inputs were slow rate modulated or bursting. However, along the sagittal axis, the stimulus driven correlations in the GrCs became strong only with simultaneous bursting mossy fiber inputs. On the other hand, input by the ascending axons of GrCs [12] was found decrease synchronization of GoCs along the parallel fiber beam because they are highly local.

Additionally, the coherence of granular layer network oscillations was affected by tonic inhibition, which is present only in the GrCs [57,58], without any effect on the oscillation frequency. Extra-synaptic GABA_A_ receptors that mediate tonic inhibition are known to be involved in many neuro-psychiatric disorders and also in memory and cognition [57], such as hippocampus-dependent learning and memory [67]. In the cerebellum, tonic inhibition improves the representation of sensory information in granule cells [68], whereas it is unclear how it affects motor learning [69].

In experiments, network oscillations in the granular layer have been probed by the LFP (reviewed in [40]). Our simulation can be augmented by recently developed softwares to compute the LFP directly [70] or via hybrid schemes [71–73], to predict how the LFP signal depends on physiological factors, which can be verified in extracellular recording experiments [74].

### Spatiotemporal structure of GrC responses to mossy fiber inputs

GrC population activity is characterized by two distinctive patterns at two time scales. On long time scales, there is an on-beam inhibition effect due to global inhibition of the unstimulated GrCs along the parallel fiber axis, which implements a *first-take-all* type mechanism. On shorter time scales, the GrC activities are regulated by the time window imposed by the timing of synchronized GoC spikes, which can regulate precision in timing, particularly regarding different latencies in the onsets of mossy fiber inputs. This is in contrast to a recent modeling study [38] that showed a much smaller inhibitory surround (< 100 μm in diameter) around an excited center. However, since that model was limited in space and had few parallel fiber contacts per GoC (~100), this was probably due to limitation of the model size. A recent in vitro study [75] also suggested that GoCs provide fast feedback inhibition to GrCs, based on the observation that a GoC receives inputs mostly from nearby GrCs but also some input from distant GrCs. Parallel fiber synapses may deliver much smaller input to a GoC soma compared to ascending axon synapses [12]. However, it has been observed that weak common inputs to individual cells can lead to robust synchronization, not only in the cerebellar network [14] but in many contexts [76]. Furthermore, our model predicts that the earliest GoC inhibition should dominate and this coincides with a recent experimental observation that the majority of GrCs receive early, not late, GoC inhibition [77].

We also observed that NMDA receptors in the GrCs play an important role by inducing a long-term increase in the GrC output gain after (burst) spiking, even in the presence of the lower voltage-dependence due to their GluN2C subunits [54]. Therefore, the GrCs that have already fired upon early mossy fiber inputs can integrate subsequent inputs much better than other cells in the network, which we called the *winner-keep-winning* mechanism. NMDA receptors have been well known for their role in supralinear synaptic integration in many systems including GrCs [56], which can contribute to information gating (e.g., [78]). The winner-keep-winning mechanism is a combined effect of two phenomena due to NMDA receptors, sustained depolarization [56] and voltage dependent synaptic integration [79], that gives an additional advantage (long-term gain upregulation) to GrCs that respond to bursting inputs. Furthermore, NMDA receptors in GrCs are known for their roles in synaptic plasticity. It has been proposed that this plasticity can tune the relative latency between the GrC firing and mossy fiber input, which in turn dictates whether the GrC firings can pass the time window imposed by the GoC feedforward inhibition[62,80].

All the mechanisms that we have discussed, the network mediated *first-take-all* and the cell intrinsic property based *winner-keep-winning*, give a predominant advantage to the GrCs that are activated earlier by the mossy fiber inputs while the others are suppressed. While this leads to a sparse spatial organization of the granular layer output, the activity within activated regions of the GrC can be quite dense due to the high dynamic range of the GrC population. This pattern of activation is compatible with the described fractured somatotopy of tactile inputs in crus II of the cerebellum [46,47]. The response to the two-patch configuration can be considered a simulation of patches activated by the same tactile input. Moreover, the larger amplitude of responses observed to the late input from sensory cortex, compared to the preceding trigeminal input [46], could be explained by the NMDA mediated increase of the GrC gain if the respective mossy fibers synapse onto the same GrCs. Note, however, that our two patch simulation results also apply to co-activated mossy fiber inputs carrying different modalities [81].

In Marr’s pioneering theoretical work [27] and following studies [30], the GoCs also play the role of regulating how many GrCs activate, but the spatiotemporal aspect of GoC firing has been largely ignored. In the mushroom body in the insect olfactory system, the synchronous and oscillatory firing of inhibitory interneurons maintain sparse firing of excitatory neurons [82]. In our model, the GoCs are governed by a similar principle since they oscillate, discharge synchronously over an extended spatial scale, and impose a narrow time window leading to effectively inhibiting a large number of GrCs, contributing to strongly spatially restricted activation. However, contrary to insect olfaction and to Marr’s theory [25], our model predicts that GrC activity within the activated patch depends on the strength of the mossy fiber stimulus and is often not sparse.

The nature of coding by the granular layer has been actively debated: Jörntell and colleagues in their study in C3 zone of decerebrated cats have found little evidence of sparse coding [20,21,83]. In C3 zone of cats, the authors reported that GrCs receive similar kind (unimodal) of mossy fiber inputs [20], whereas diverse mossy fiber inputs should converge at a GrC (multimodal) for sparse coding to work effectively. Also, GrCs in their study were not silent at rest and fired a barrage of spikes in response to peripheral activation [20]. However, other studies in mouse cerebellar cortex demonstrated that GrCs receive multimodal mossy fiber input. For example, Huang *et al* reported convergence of proprioceptive (external cuneate nucleus) and pontine (basilar pontine nucleus) inputs in various regions of the cerebellar cortex [84]. Convergence of multimodal mossy fiber inputs (vestibular, visual) is found in the GrCs of the vestibulocerebellum [85] and in the hemispheres (tactile, auditory and visual) [81]. Our model is neutral towards the convergence discussion because we did not specify what information is carried by the mossy fiber input.

The sparse coding by the GrCs hypothesis has recently also been challenged based on experimental observations of dense coding by GrCs [36] and that the GrCs also rate code the rate modulated MF inputs [83,86] (see also [87]). Similarly, our model showed that, despite strong temporal patterning by the GoCs, the GrC population rate follows the rate modulated mossy fiber input quite well, resulting in a large dynamic range. Furthermore, spatially separated GrC populations can co-activate, when each of them are stimulated by a different mossy fiber group. Note that this would be impossible if GoC inhibition is purely based on an asynchronous rate code, since no time window for co-activation would be allowed. Therefore, the rich spatiotemporal dynamics of our model provides a unified viewpoint for the resolution of experimental controversies about coding in the cerebellar granular layer.

### Conclusion

Our simulations suggest that oscillations characterize the basic network activity of cerebellar granular layer network along with stochastic spiking of GoCs and GrCs and rich spatio-temporal dynamics. A *first-take-all* mechanism based on the network structure and NMDA receptor mediated *winner-keep-winning* mechanisms further characterize the spatiotemporal dynamics of granular cell firing. Wide dynamical range indicates a large flexibility in the allocation of granule cells, ranging the encoding from sparse to dense. Based on our results, we suggest that the unique anatomy of the cerebellar granular layer, coupled with cellular and network mechanisms promote spatial group selection of GrC activity as a function of MF input timing and spatial organization.

## Methods

All simulations were carried out using the NEURON simulation platform (version 7.4) [88] on the OIST high-performance computing cluster, running on 200 cores. The mean time taken to run a “biological millisecond” was 3.00±0.03 seconds. The model is made publicly available at ModelDB (http://senselab.med.yale.edu/modeldb) under the accession number 232023.

### Single cell models

We used previously published models of GrCs and GoCs [38] except that the dendritic morphology of a GoC was modified: two shorter (60 μm long) baso-lateral dendrites were constrained to the granular layer and the other two, longer (~166 μm long), apical dendrites extended into the molecular layer as in [1]. We also reduced the diameter of the dendrites to 2.4 μm to match the electrical and firing properties to the original model. All the cell and synapse models (see below) were simulated at the temperature of 37°C. For simulations with tonic inhibition, we included a tonic conductance of 88 pS with a reversal potential at -73 mV in a GrC model, which resulted in ~260 pS of total tonic conductance [32], which includes stationary activation of GABAergic synapses in the baseline condition.

### Network architecture

Our granular layer network model is based on detailed anatomical information previously published [1,4,5,7,16,18,19,31,35,89,90]. The 3D network model has dimensions of 1500 μm along the transverse axis, 700 μm along the sagittal axis and 430 μm along the vertical axis (Fig 1). The granular and molecular layers were 200 μm thick each, with a 30 μm thick Purkinje cell layer between them. The number of neurons in the network was determined in the following way: We first calculated the number of GoCs in the network using the anatomical GoC density (9500 cells/mm^3^) [18]. From this we calculated the number of GrCs in the network using the GrC to GoC ratio [4]. The total number of GoCs in the network for the above-mentioned network dimension was 1,995 and total number of GrCs amount to 798,000. The somatic centers of all the neurons were uniformly distributed in the granular layer.

After this, we determined the connectivity between the neurons based on connectivity rules that we will explain in the following section. The neurons were then connected with experimentally validated synapses and gap junctions with corresponding conduction delays depending on their mutual distances. The conduction velocity of parallel fiber axons was set to 0.3 m/s [91,92], while that of mossy fiber and GoC axons was 2 m/s [93].

### Mossy fibers and rosettes

Mossy fiber rosette distribution was based on that of LRN mossy fiber axons [7]: Rosettes of a single primary collateral of LRN axon distribute widely along the parasagittal axis, but along the transverse axis the spread is limited. As a result, rosettes of a single LRN axon are arranged in sagittal strips parallel to each other along the transverse axis. A similar parasagittal arrangement of mossy fiber rosettes is also reported in other studies [35].

We first constructed mossy fibers with a density of 5000 fibers/mm^2^, which is based on the projection density of mossy fibers in C1 zone of Paramedian lobule of the cerebellum [94,95]. Due to network size limitations, the distribution of mossy fiber rosettes in the model is based on that of a single primary collateral of a LRN axon [7]. For each mossy fiber in the model, the rosettes were distributed according to the rosette cluster distribution of primary collaterals of LRN axon [7]. We used another experimental data set about the distribution of pontine mossy fiber rosettes ([96], private communication with Daria Rylkova) to optimize their distribution in the model. For each mossy fiber in the model, we adjusted the extent of spread of rosettes both along the long axis and sagittal axis until the amount of overlap between mossy fibers closely matched that of the pontine mossy fiber data. We measured the amount of overlap between different mossy fibers in the model and experimental (pontine) data as follows: We divided the entire volume into a number of small cubes and counted the number of distinct mossy fibers represented in each cube. From this data, we calculated the relative number of cubes representing 0,1,2,3 and 4 distinct mossy fibers. This was then repeated for different cube sizes. We computed the final mossy fiber density using anatomical ratio of glomerulus to GrC [31]. In order to eliminate boundary effects, we also instantiated mossy fibers around the network when the rosettes projected into the model. Total number of mossy fibers that project at least one rosette into the model was 2109 and total number of rosettes was 29519.

### Network connectivity

The connectivity between neurons in the network is based on anatomical connectivity patterns observed in the cerebellar granular layer [5,31,89,97,98]. The model has synapses projecting from excitatory mossy fibers to GrCs, mossy fibers to GoCs, inhibition by GoCs of GrCs, excitation by GrCs of GoCs through ascending axons and parallel fibers (Fig 2). In addition to the synapses listed above, GoCs are connected by gap junctions [16,18] and inhibitory synapses [19]. Convergence, divergence and synaptic parameters for each synapse in the model are described in S1 Table.

Except for synapses in GrCs (see below), the time course of synaptic conductance G_syn_(t) was modeled according to the standard double exponential equation [99]

$$G_{syn}(t)=gmax\times N\times \left[exp\;(\frac{-t}{\tau_{decay}})-exp\;(\frac{-t}{\tau_{rise}})\right]$$ (1)

where τ_rise_ and τ_decay_ are rise and decay time constant respectively. *gmax* is peak synaptic conductance, and N is a normalization factor that makes the maximum of G_syn_(t) equal to *gmax*. τ_rise_ and τ_decay_ were obtained by fitting Eq 1 to the respective experimental traces.

### Mossy fiber to GrC connectivity

The connectivity between mossy fibers and GrCs is based on the maximum length of the GrC dendrite [31]. For each GrC, we formed a sphere of radius of 30 μm around its center and connected to rosettes within that sphere in a probabilistic manner. On average, each GrC received 4.5±1.5 (2–7) distinct mossy fiber connections.

We used the mossy fiber-to-GrC synapse model of [38] (deterministic version) with the following modifications: First, neurotransmitter diffusion is approximated by a cascade linear process [100],

$$\frac{dP}{dt}=-r_{fast}P,$$

$$\frac{dT}{dt}=-r_{T}T+P-r_{T1}(T-I_{1}),$$

$$\frac{dI_{1}}{dt}=r_{1T}(T-I_{1})-r_{12}(I_{1}-I_{2}),$$

$$\frac{dI_{2}}{dt}=r_{21}(I_{1}-I_{2})-r_{23}(I_{2}-I_{3}),$$

$$\frac{dI_{3}}{dt}=r_{32}(I_{2}-I_{3}),$$ (2)

where *T* is the concentration of diffused neurotransmitter. At each presynaptic spike, *P* is transformed as *P*→*P+y* where *y* represents a diffusing fraction of released neurotransmitter, controlled by a synaptic facilitation/depression mechanism. The parameters are given as *r*_*fast*_ = 4/*τ*_*D*_, *r*_*T*_ = 6.2/*τ*_*D*_, *r*_*D*1_ = 20/*τ*_*D*_, *r*_1*D*_ = 9.09/*τ*_*D*_, *r*_12_ = 4.9/*τ*_*D*_, *r*_21_ = 1.71/*τ*_*D*_, *r*_23_ = 0.55/*τ*_*D*_, and *r*_32_ = 0.333/*τ*_*D*_. The diffusion time constant *τ*_*D*_ is given by $\tau_{D}=100\;R_{d}^{2}/4D$ where *R*_*d*_ = 1.03 μm and *D* = 4 μm^2^/ms [38,79]. This scheme provided a good approximation of the AMPA and NMDA activation over a wide range of presynaptic inputs (S6A Fig). Second, we set the desensitization constant of the NMDA receptor to 12×10^−4^ ms^-1^ [79]. Finally, the voltage dependence of the NMDA receptors is modeled as

$$f(V)=\frac{1}{1+\frac{{\left[Mg\right]}_{o}}{K_{Mg}}exp\left(-V/\delta_{V}\right)}$$ (3)

where [Mg]_o_ = 1 mM, *K*_Mg_ = 1.77 mM, and *δ*_*V*_ = 22.4 mV [54].

Conductance parameters of the receptors were adjusted to have GrC firing ~1 Hz in the baseline condition (mossy fiber firing at 5 Hz) and also its ~6 fold increase in the absence of inhibition [68].

### Mossy fiber to GoC connectivity

For connectivity between mossy fibers and GoCs, we assumed a sphere of radius 100 μm and connected GoCs and rosettes within that sphere probabilistically. Each GoC in the model received an average of 13.7±6.5 (1–36) distinct mossy fiber connections. The mossy fiber to GoC synapse is glutamatergic (only AMPA receptors) whose synaptic parameters were obtained from the experimental recordings of GoC EPSCs [101].

### GoCs to GrCs connectivity

Inhibitory connections between GoCs and GrCs were based on the extent of axonal arborization of the former. GoC axons exhibit a parasagittal organization (Fig 2) [5]. Distribution of their axonal boutons is about 650 μm along the parasagittal axis and about 180 μm along the medio-lateral axis. We assumed a connection probability that generated 8.4±3.2 (1–22) GoC synapses per GrC on average.

Synaptic parameters were based on experimental data [13] and included an indirect spillover component (S6B Fig). The IPSC decay consisted of two components: the transient component with a time constant of 5 ms and an indirect spillover component with a time constant of 35 ms (that contributed to 10% peak amplitude). The IPSC rise time constant was 3 ms.

### Parallel fiber/ascending axon to GoC connectivity

Connections from GrCs to GoC via parallel fibers/ascending axons were generated using our custom tool, the Boundary Representation Language (BREP) [102]. In this method, the geometric structures associated with the connectivity (parallel fibers/ascending axon and GoC apical dendrites) were described as points in space along a straight line in three dimensions. The ascending axon of each GrC was modeled as a straight vertical line of length 200 μm with points separated by 50 μm. The parallel fibers of each cell were modeled as two straight lines of length 1000 μm each (extending on either side of their bifurcation from the ascending axon in the molecular layer) with points separated by 7.5 μm. Small random perturbations were added to both.

GoCs also had random angular displacements of their dendritic points. In each GoC, the dendritic elements were modeled as lines that lie on the surface of an inverted cone of height 332 μm (apical dendrite) or 6 μm (baso-lateral dendrite). Each dendritic element was created with a randomly chosen angle from a normal distribution with mean (30°, 120°) for apical and (-20°, -240°) for basolateral and standard deviation of 10°. The elements were first rotated on to the circumference of a circle of radius 100 μm and raised (apical in molecular layer) or lowered (basolateral in granular layer) thereby forming an inverted cone. The GoC axons were represented as uniformly distributed random points in a rectangular area (boundaries in μm: transverse [-45:45], sagittal [-160:160], vertical [-75:75]) relative to the soma position.

Once the points associated with each geometric cell structure were generated, we used a K-d tree data structure [103] to order the points and performed fast nearest neighbor searches. We assumed a connectivity radius of 30 μm and 5 μm for ascending axon and parallel fiber connections, respectively. The connectivity probability was chosen to achieve the target number of connections. On the average, each GoC in the model received about 554±302 (55–1245) ascending axon connections. The number of parallel fiber synapses (4759±1037 (2512–6582)) on a single GoC in the model was calculated based on the density of parallel fiber synapses in the molecular layer [98] and also based on the fact that approximately 9% of them are formed on structures other than Purkinje neuron spines [97]. Both ascending axon and parallel fiber synapses on GoC dendrite are AMPAergic with time constants and maximal synaptic conductance described in S1 Table.

### GoCs to GoCs inhibitory connectivity and gap junctions

We connected the GoCs with gap junctions [16,18] and inhibitory connections [19]. Inhibitory and gap junctional connectivity between GoCs were also generated by BREP. The probability distribution function (Boltzmann function) for gap junction connectivity was based on an experimental published data [16] and the conductance decayed as a function of distance [18] as *g* = *β* exp(-*λx*) where *β* = 1.659 nS and *λ* = 0.01259 μm^-1^. Each GoC had about 13.7±4.6 (1–31) gap junctions on average. For inhibitory connections between the GoCs, we used the experimental measurements (20% connectivity probability at 50 μm) from ref. [19], coupled with the gap junction connection probability data. Each GoC received inhibitory input from 2.2±1.6 (0–10) GoCs on average.

### Modeling extra inhibitory inputs to GoCs

As we tuned the model with the background mossy fiber firing of 5 Hz by varying synaptic conductances, we discovered that the firing rate of GrCs and GoCs tended to covary with a ratio of GrC:GoC ≈ 1:30. This suggested that GoCs needed extra inhibitory inputs to reproduce *in vivo* observations of GrC:GoC ≈ 1:8–18. Recent studies also suggested that the inhibitory inputs mostly originate from extracortical neurons [50,51], which are not in our model.

To simulate the effect of those inhibitory inputs, we included tonically active GABA receptors in the apical dendrites of the GoCs. We estimated that they should roughly correspond to ~150 synapses with a peak conductance ~180 pS [51] and also a 5 ms decay constant in *in vivo*-like conditions. A range of activation rates from 15 to 20 Hz robustly resulted in ~1 Hz and ~10 Hz firing of GrCs and GoCs, respectively, with a 5 Hz mossy fiber input, and we chose 16 Hz, which led to the resulting total conductance of 2160 pS.

### Randomization

We introduced random variations in the cellular and synaptic parameters as follows: GoC and GrC soma diameters were randomly varied by up to 20%, and their initial resting membrane potential was also varied in the range -60 to -75 mV. For each type of synapse, the peak conductances were varied in a manner so that they had a coefficient of variation of 0.25.

### Network stimulation paradigms

We generated firing of each mossy fiber by using a leaky integrate-and-fire (LIF) neuron model driven by a noisy current input: the membrane voltage of the model was given by

$$\frac{dV}{dt}=-\frac{V-E}{\tau }+\beta \mu (t)+\sigma (t)\xi (t),$$

where *τ* = 1 ms, *E* = -70 mV and *ξ*(*t*) was a Gaussian white noise updated every 1 ms. The spike threshold was at *V* = -60 mV. After a spike, a refractory period of 1.1 ms was imposed and then *V* was reset to *E*. *μ*(t) and *σ*(t) were controlled by a common parameter *ν*(*t*) as *μ*(*t*) = *gNν*(*t*) and $\sigma (t)=g\sqrt{N\nu (t)}$ where *N* = 1000 and *g* = 5 μV/ms. We chose *β* = 0.01 to ensure that the model would fire mostly due to noisy fluctuations in the input. We first generated a table of constant *ν* vs. the output firing rate, and used it to calculate *ν*(*t*) for a certain target firing rate by linear interpolation.

The background firing of each unstimulated mossy fiber was generated by the LIF neurons firing at 5 Hz as in the *in vivo* recordings [32]. The stimulated mossy fibers were activated in patches of 100 or 200 μm radius, with different input patterns such as slow rate modulation or bursting. In the slow rate modulation paradigm, the input was defined by upper and lower bound frequencies, each lasting 300 ms. The lower bound frequency was kept constant at 10 Hz for all simulations. Upper bound frequency was varied between 50–60 Hz. A combination of an upper bound and lower bound is an epoch, which lasts for 600 ms and each of the rate modulated mossy fiber stimuli consists of five epochs (Fig 5A). The firing rate epochs were smoothed with a Gaussian kernel (σ = 50 ms) and the LIF spike trains were generated based on the rate. In the burst input paradigm, we activated mossy fibers in patches of 100 μm with bursts of frequency 500 Hz and duration 10 ms.

Mossy fibers were activated in single or two patches either along the parallel fiber axis or sagittal axis. For simulations involving ascending axon mediated de-synchronization of GoCs along the transverse axis, we eliminated the difference in temporal structure in mossy fiber input between the two activated patches (patch 1 and 2) in the following way: For each GoC in patch 1, a corresponding GoC was randomly picked (without replacement) from patch 2. Mossy fiber connectivity to the GoC from patch 2 was made identical to that of the GoC from patch 1.

### Data analysis

We recorded spike times of all neurons during the course of simulation. Simulations were repeated 5 times with different global random seeds, which also affected the network structure. Data was then analyzed using MATLAB version R2011b (Mathworks, MA, USA) software. Spike times were transformed into spike trains with 1 ms long time bins. We often evaluated the average activity of specific neurons within a certain region by taking the average of the corresponding spike trains.

Oscillations were measured by binning the spikes of the population (GoC or GrC) in 1 ms long time bin. Power spectral density of the resulting oscillations was calculated and oscillation frequency was taken as the frequency corresponding to peak power in the power spectral density. The synchronization index for GoC oscillations was calculated as the proportion of total number of GoCs involved in each oscillatory cycle, calculated by integrating the area under each oscillatory cycle. The firing rate of ON patch neurons in Figs 9, 10 and 11 was computed for a time period of 30 ms from the burst onset. For Figs 6 and S2 (slow rate coded input), the firing rate of ON patch neurons was computed for a period of 100 ms (during the upstroke of the epoch when mossy fiber firing rate is maximum). The firing rate of OFF patch neurons was computed during the corresponding time period. All indicated values in the study represent mean ± standard deviation.

Dynamic range of GC activation (Fig 4) was quantified for both ON patch and OFF patch GrCs (both transverse and sagittal axis) when simulated with different mossy fiber firing rates. This was done by calculating the percentage of active GrCs in different time windows (1,10,100 ms) over the course of the entire simulation and averaging it across different data sets. Dynamic range was then calculated using the formula, $Dynamic\;range=\frac{max\;value}{min\;value}$.

### Volumetric maps and movies

The volumetric reconstructions of cell activity are achieved by binning the cells spiking within any millisecond of simulation in a voxel of 10 μm. This produces a 3D histogram of spike counts per voxel. This volume is then convolved with 3D Gaussian kernels normalized by the maximum value of the kernel. The resulting voxel value is proportional to the maximum number of cells active in the voxel. The result is passed as color and alpha components to the MATLAB function Vol3D (http://www.mathworks.com/matlabcentral/fileexchange/22940-vol3d-v2).

### Cross-correlation

Cross-correlations were computed between the average activities of neurons in two selected patches, regions of 100 or 200 μm radius in the model. We first formed the spike trains of all the neurons with 1 ms time bins. The average activity *y*_*A*_ for patch *A* is given by,

$$y_{A}=\frac{\left(\sum_{i=1}^{N_{A}}A_{i}\right)}{N_{A}}$$ (4)

where *N*_*A*_ is the number of the GrCs or GoCs in *A*.

The cross-correlation function (CCF) between region *A* and *B* is given by

$$CCF_{AB}\;(t)=\frac{1}{{L\times Z}_{AB}}\sum_{s=1}^{L-t}(y_{A}(s+t)–\bar{y_{A}})\times {(y}_{B}(s)-\bar{y_{B}})\;if\;t\geq 0,$$

$$CCF_{AB}\;(t)=\frac{1}{{L\times Z}_{AB}}\sum_{s=1-t}^{L}(y_{A}(s+t)–\bar{y_{A}})\times {(y}_{B}(s)-\bar{y_{B}})\;if\;t<0,$$ (5)

where $Z_{AB}=\sqrt{\left(Var[y_{A}]\times Var[y_{B}]\right)}$ and *L* is the length of *y*_*A*,*B*._

CCF was computed for t = ±300 time lags.

We defined cross-correlation ‘*c*’ as oscillatory synchrony after discounting the effect of firing rate modulation. We denote the measured correlation coefficient by ‘*a’* (zero-time lag correlation CCF (*t* = 0)), the expected coefficient from firing modulation only by ‘*b’*, and compute c as *c = a-b*. In all sections of the results we report the value of *‘c’* as cross-correlation. To find the effect of firing rate co-modulation, the average population activities for each patch were low pass filtered below 10 Hz, which was above the frequency range of our input firing rate modulation. We computed CCFs based on them according to Eq 4, except that the normalization factor *Z*_*AB*_ is still based on the unfiltered spike trains. This scheme made it easier to compare cross-correlations at two different time scales (e.g., blue and red lines in Fig 5D–5G).

Statistical significance of CCF (*t* = 0) is non-parametrically evaluated by counting the number of the outliers *n*_*out*_ in {CCF_shuffled_} whose amplitude exceeded that of CCF (*t* = 0). CCF_shuffled_ was computed for *t* = ±300 time lags in the following way: In each of the patches, we divided each simulation epoch, which can be the period of the entire simulation or each stimulation protocol depending, into a number of small sub-epochs (of length 10 ms) and randomly shuffled the GrC and GoC spike trains in the divided sub-epochs. This gave us the average activity *z*_*A*_ and *z*_*B*_ from the shuffled spike trains of two patches. *N*_*shuffle*_ was chosen to be 50. CCF_shuffled_ was calculated from *z*_*A*_ and *z*_*B*_ according to Eq 4. Then, the p-value of CCF(*t*) was estimated by an empirical type-I error rate, $p=\frac{n_{out}}{N_{total}}$, where *N*_*total*_ = *N*_*shuffle*_ × (2 × *nlag* + 1). We assumed a confidence interval of 99% and *p*<0.01 was considered to be significant.

We also tried to increase the statistical power by combining the results from multiple simulations. In this case, CCFs were appropriately averaged and the p-values were obtained from combined observations. Error bars in cross-correlation were obtained by bootstrap resampling.

## Supporting information

S1 Table Convergence, divergence, total number of synapses and synaptic parameters for various synapses in the network. (DOCX)

S1 Fig Membrane potential traces of Golgi and granule neurons for various stimulation paradigms. **A-D**: Membrane potential traces of ON patch Golgi (blue) and granule neurons (red) when stimulated with 60 Hz Poisson input.
**E-H**: Same as A-D for slow rate modulated input.
**I-L**: Same as A-D for burst input.
(TIFF)

S2 Fig Firing rate and cross-correlation along the sagittal axis. **A-D:** GoC firing rate (A), cross-correlation (B), GrC firing rate (C), cross-correlation (D) along the sagittal axis when the network was activated with a single patch of mossy fibers with slow rate coded input. Black lines represent background network firing rate for the respective patches.
**E-H:** Same as A-D with two ON patches separated by 400 μm along the sagittal axis whose mossy fiber input rate is modulated identically.
**I-L:** Same as E-H, while rate modulation in two patches are different as in Fig 6I–6L.
Asterisk and triangle represent significant (p<0.01) and insignificant correlation (p> = 0.01), respectively. The stimulation and recording configuration shown as insets in D, H, and L follows the same scheme as in Fig 6. Data are mean±standard deviation.
(TIFF)

S3 Fig Volumetric maps of network activity in response to slow rate coded input activated in two patches along the transverse axis. A: GrC population PSTH showing the timing of volumetric maps at various points during the network activity.
B-G: Volumetric maps that portray the network activity at various time points.
The stimulus onset is at 0 ms. One can see the patchy mossy fiber activation, GrC spiking activity in response to that and GoC activity all along the transverse axis.
The colors represent the same scheme as explained in S1 Movie.
(TIFF)

S4 Fig Volumetric maps of network activity in response to single burst input activated in a single patch at the center of the network. A: GrC population PSTH showing the timing of volumetric maps at various points during the network activity.
B-G: Volumetric maps that portray the network activity at various time points.
The stimulus onset is at 0 ms. One can see the mossy fiber burst, GrC bursting activation, GoC activity all along the transverse axis. Network rebound response can also be seen from panel G. The colors represent the same scheme as explained in S1 Movie.
(TIFF)

S5 Fig Firing rate and cross-correlation along the sagittal axis with bursting mossy fiber input. **A-D:** GoC firing rate (A), cross-correlation (B), GrC firing rate (C), cross-correlation (D) along the sagittal axis when the network was activated with a single patch of mossy fibers with bursting inputs. Black lines represent background network firing rate for the respective patches.
**E-H:** Same as A-D with two ON patches separated by 400 μm along the sagittal axis with bursting mossy fibers inputs.
Asterisk and triangle represent significant (p<0.01) and insignificant correlation (p> = 0.01), respectively. The stimulation and recording configuration shown as insets in D and H represent the same configurations as those in Fig 6.
(TIFF)

S6 Fig Models of dynamic synapses on GrCs. **A:** Black lines are the AMPA and NMDA current induced by presynaptic spikes (blue) in the original model [79]. Red lines are the same currents in our model where the glutamate diffusion is approximated by a cascade linear process (Eq 2).
**B:** An experimental data of GrC eIPSC copied from [13] (black), and eIPSC of our GABAergic synapse model in the same condition (red).
(TIFF)

S1 Movie Video of network activity in response to 60 Hz patchy mossy fiber input activated in a single patch at the center of the network. The blue and green dots represent mossy fiber and GoC activity respectively. The red points represent granule cell activity. The stimulus onset is at 0 ms. One can see the patchy mossy fiber activation, GrC spiking activity in response to that and GoC activity all along the transverse axis.
(MP4)

S2 Movie Video of network activity in response to slow rate coded input activated in two patches along the transverse axis. The colors represent the same scheme as explained in S1 Movie.
(MP4)

S3 Movie Video of network activity in response to burst input activated in a single patch at the center of the network. The colors represent the same scheme as explained in S1 Movie.
(MP4)
