# Correction: Spatiotemporal network coding of physiological mossy fiber inputs by the cerebellar granular layer

**DOI:** 10.1371/journal.pcbi.1007472

**Published:** 2019-10-22

**Authors:** Shyam Kumar Sudhakar, Sungho Hong, Ivan Raikov, Rodrigo Publio, Claus Lang, Thomas Close, Daqing Guo, Mario Negrello, Erik de Schutter

There is an error in the Funding statement. The grant number is incorrect and should be 15K06725 instead of 15K06715.

The full correct statement should read: “This work was supported by funding from the Okinawa Institute of Science and Technology Graduate University. The funders had no role in study design, data collection and analysis, decision to publish, or preparation of the manuscript. SH was supported by the Japan Society for the Promotion of Science, KAKENHI Grant Number 15K06725 (https://www.jsps.go.jp/english/e-grants/).”
