# Supplementary material for: Spatiotemporal network coding of physiological mossy fiber inputs by the cerebellar granular layer
Source: PLoS Comput Biol. 2017 Sep 21;13(9):e1005754. doi: 10.1371/journal.pcbi.1005754 (PMC5626500; doi:10.1371/journal.pcbi.1005754)
Supplement: S1 Table — (DOCX) [file pcbi.1005754.s001.docx]

| **Connectivity name** | **Convergence** | **Divergence** | **Total connections** | **Synaptic parameters** | **References** |
| --- | --- | --- | --- | --- | --- |
| MF-GrC (AMPA) | 4.5 | 1640.6 | 3426561 | *g*_AMPA_ = 900 pS,  other parameters are the same as [1] | [2,3,1] |
| MF-GrC (NMDA) | 4.5 | 1640.6 | 3426561 | *g*_NMDA_ = 12690 pS, *R*_desenstize_ = 1.2 s^-1^, other parameters are the same as [1] | [2,3,1] |
| MF-GoC  (AMPA) | 13.65 | 12.5 | 26161 | *τ*_rise_ = 0.13 ms,  *τ*_decay_ = 1.1 ms,  *g*_max_ = 300 pS | [4,5] |
| GoC-GrC  (GABA) | 8.4 | 3364.65 | 6712206 | *τ*_rise_ = 3 ms,  *τ*_decay1_ = 5 ms, *τ*_decay2_ = 35 ms,  *g*_max_ = 100 pS | [6,7] |
| GrC-GoC (AA-AMPA) | 554 | 1.36 | 1089460 | *τ*_rise_ = 0.06 ms,  *τ*_decay_ = 0.5 ms,  *g*_max_ = 200 pS | [5] |
| GrC-GoC (PF-AMPA) | 4759 | 11.34 | 9172885 | *τ*_rise_ = 0.06 ms,  *τ*_decay_ = 0.6 ms,  *g*_max_ = 200 pS | [5,8,9] |
| GoC-GoC  (GJs) | 13.7 | 13.7 | 13132 | *g*_max_ = 1.66 nS | [10,11] |
| GoC-GoC  (GABA) | 2.2 | 2.2 | 4320 | *τ*_rise_ = 1.9 ms,  *τ*_decay_ = 14.1 ms,  *g*_max_ = 330 pS | [12] |

GrC- granule neuron, GoC-Golgi neuron, GJs-gap junctions, AA-ascending axons, PF-parallel fibers, MF-mossy fibers.

**References**

1. Solinas S, Nieus T, D’Angelo E. A realistic large-scale model of the cerebellum granular layer predicts circuit spatio-temporal filtering properties. Front Cell Neurosci. 2010;4: 12. doi:10.3389/fncel.2010.00012

2. Palkovits M, Magyar P, Szentágothai J. Quantitative histological analysis of the cerebellar cortex in the cat. IV. Mossy fiber-Purkinje cell numerical transfer. Brain Res. 1972;45: 15–29. doi:10.1016/0006-8993(72)90213-2

3. Nieus T, Sola E, Mapelli J, Saftenku E, Rossi P, D’Angelo E. LTP regulates burst initiation and frequency at mossy fiber-granule cell synapses of rat cerebellum: experimental observations and theoretical predictions. J Neurophysiol. 2006;95: 686–699. doi:10.1152/jn.00696.2005

4. Kanichay RT, Silver RA. Synaptic and cellular properties of the feedforward inhibitory circuit within the input layer of the cerebellar cortex. J Neurosci. 2008;28: 8955–8967. doi:10.1523/JNEUROSCI.5469-07.2008

5. Cesana E, Pietrajtis K, Bidoret C, Isope P, D’Angelo E, Dieudonne S, et al. Granule Cell Ascending Axon Excitatory Synapses onto Golgi Cells Implement a Potent Feedback Circuit in the Cerebellar Granular Layer. J Neurosci. 2013;33: 12430–12446. doi:10.1523/JNEUROSCI.4897-11.2013

6. Mapelli L, Rossi P, Nieus T, D’Angelo E. Tonic activation of GABAB receptors reduces release probability at inhibitory connections in the cerebellar glomerulus. J Neurophysiol. 2009;101: 3089–3099. doi:10.1152/jn.91190.2008

7. Simões de Souza FM, De Schutter E. Robustness effect of gap junctions between Golgi cells on cerebellar cortex oscillations. Neural Syst Circuits. BioMed Central Ltd; 2011;1: 7. doi:10.1186/2042-1001-1-7

8. Napper RM, Harvey RJ. Number of parallel fiber synapses on an individual Purkinje cell in the cerebellum of the rat. J Comp Neurol. 1988;274: 168–177. doi:10.1002/cne.902740204

9. Pichitpornchai C, Rawson J a, Rees S. Morphology of parallel fibres in the cerebellar cortex of the rat: an experimental light and electron microscopic study with biocytin. J Comp Neurol. 1994;342: 206–20. doi:10.1002/cne.903420205

10. Dugue GP, Brunel N, Hakim V, Schwartz E, Chat M, Levesque M, et al. Electrical Coupling Mediates Tunable Low-Frequency Oscillations and Resonance in the Cerebellar Golgi Cell Network. Neuron. 2009;61: 126–139. doi:10.1016/j.neuron.2008.11.028

11. Vervaeke K, LÖrincz A, Gleeson P, Farinella M, Nusser Z, Silver RA. Rapid Desynchronization of an Electrically Coupled Interneuron Network with Sparse Excitatory Synaptic Input. Neuron. 2010;67: 435–451. doi:10.1016/j.neuron.2010.06.028

12. Hull C, Regehr WG. Identification of an Inhibitory Circuit that Regulates Cerebellar Golgi Cell Activity. Neuron. Elsevier Inc.; 2012;73: 149–158. doi:10.1016/j.neuron.2011.10.030
