# Supplementary figures and images for: Spatiotemporal network coding of physiological mossy fiber inputs by the cerebellar granular layer
Source: PLoS Comput Biol. 2017 Sep 21;13(9):e1005754. doi: 10.1371/journal.pcbi.1005754 (PMC5626500; doi:10.1371/journal.pcbi.1005754)

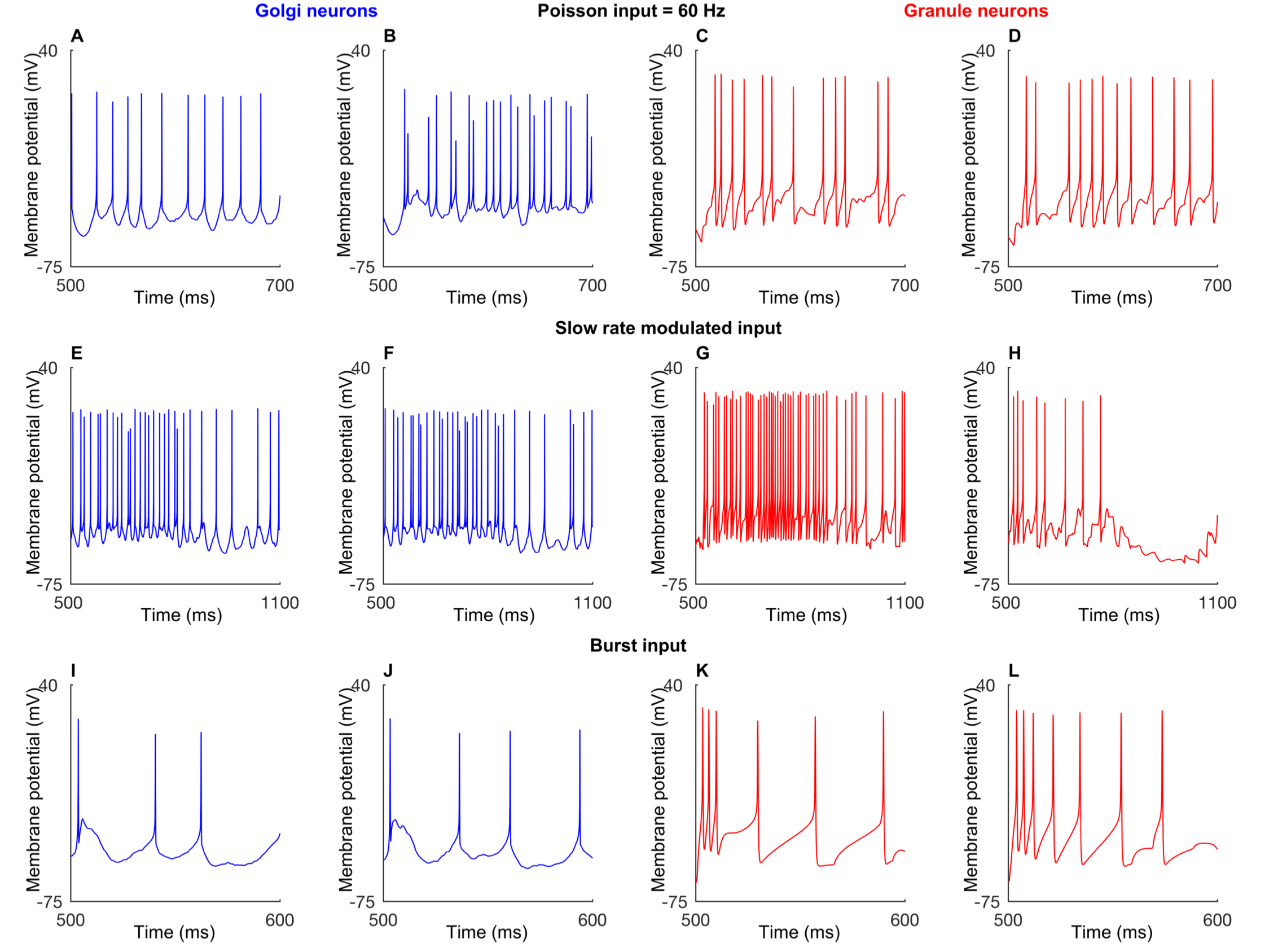

Supplement: S1 Fig — A-D: Membrane potential traces of ON patch Golgi (blue) and granule neurons (red) when stimulated with 60 Hz Poisson input. E-H: Same as A-D for slow rate modulated input. I-L: Same as A-D for burst input. (TIFF) [file pcbi.1005754.s002.tiff]

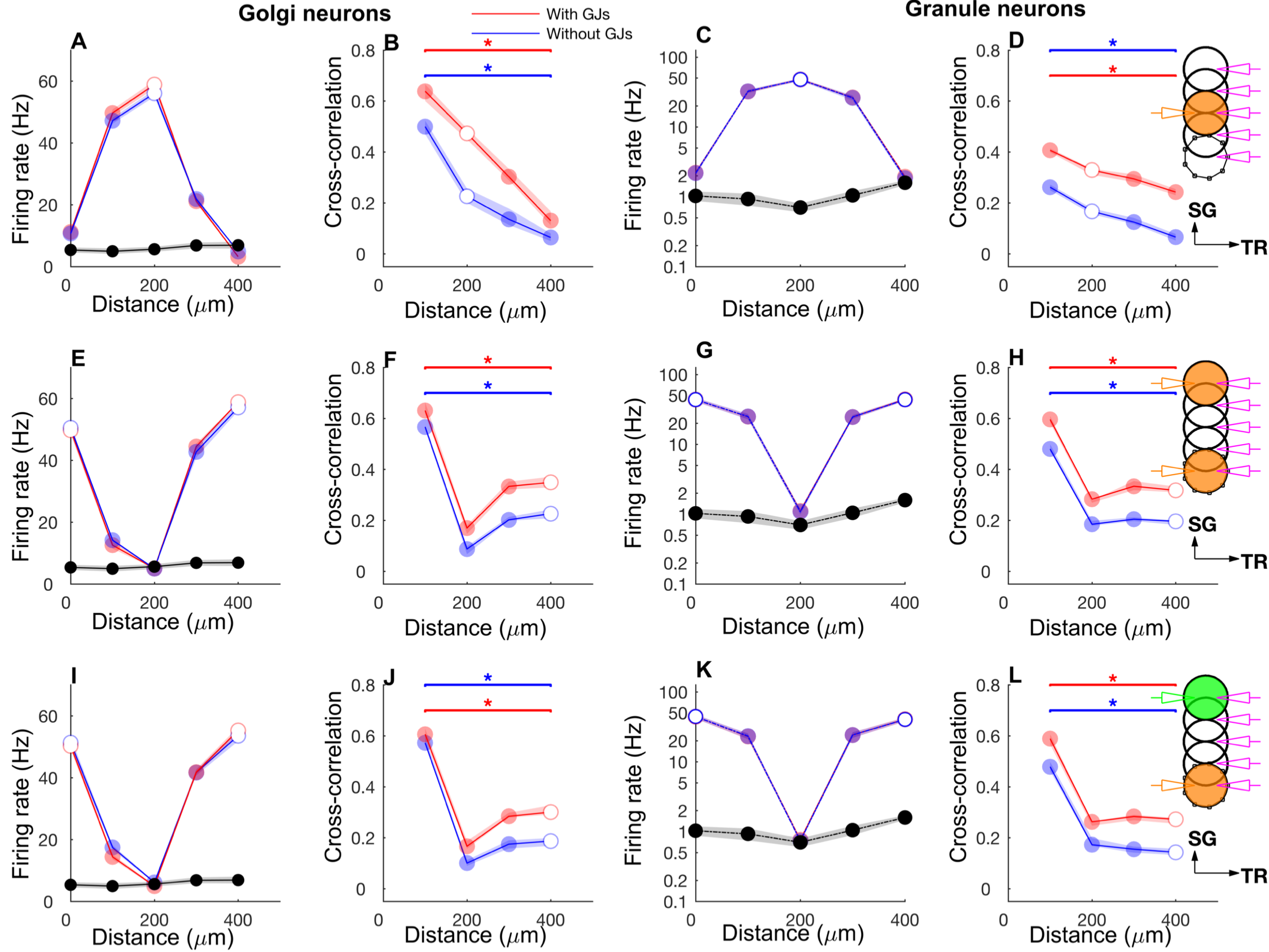

Supplement: S2 Fig — A-D: GoC firing rate (A), cross-correlation (B), GrC firing rate (C), cross-correlation (D) along the sagittal axis when the network was activated with a single patch of mossy fibers with slow rate coded input. Black lines represent background network firing rate for the respective patches. E-H: Same as A-D with two ON patches separated by 400 μm along the sagittal axis whose mossy fiber input rate is modulated identically. I-L: Same as E-H, while rate modulation in two patches are different as in Fig 6I–6L. Asterisk and triangle represent significant (p<0.01) and insignificant correlation (p> = 0.01), respectively. The stimulation and recording configuration shown as insets in D, H, and L follows the same scheme as in Fig 6. Data are mean±standard deviation. (TIFF) [file pcbi.1005754.s003.tiff]

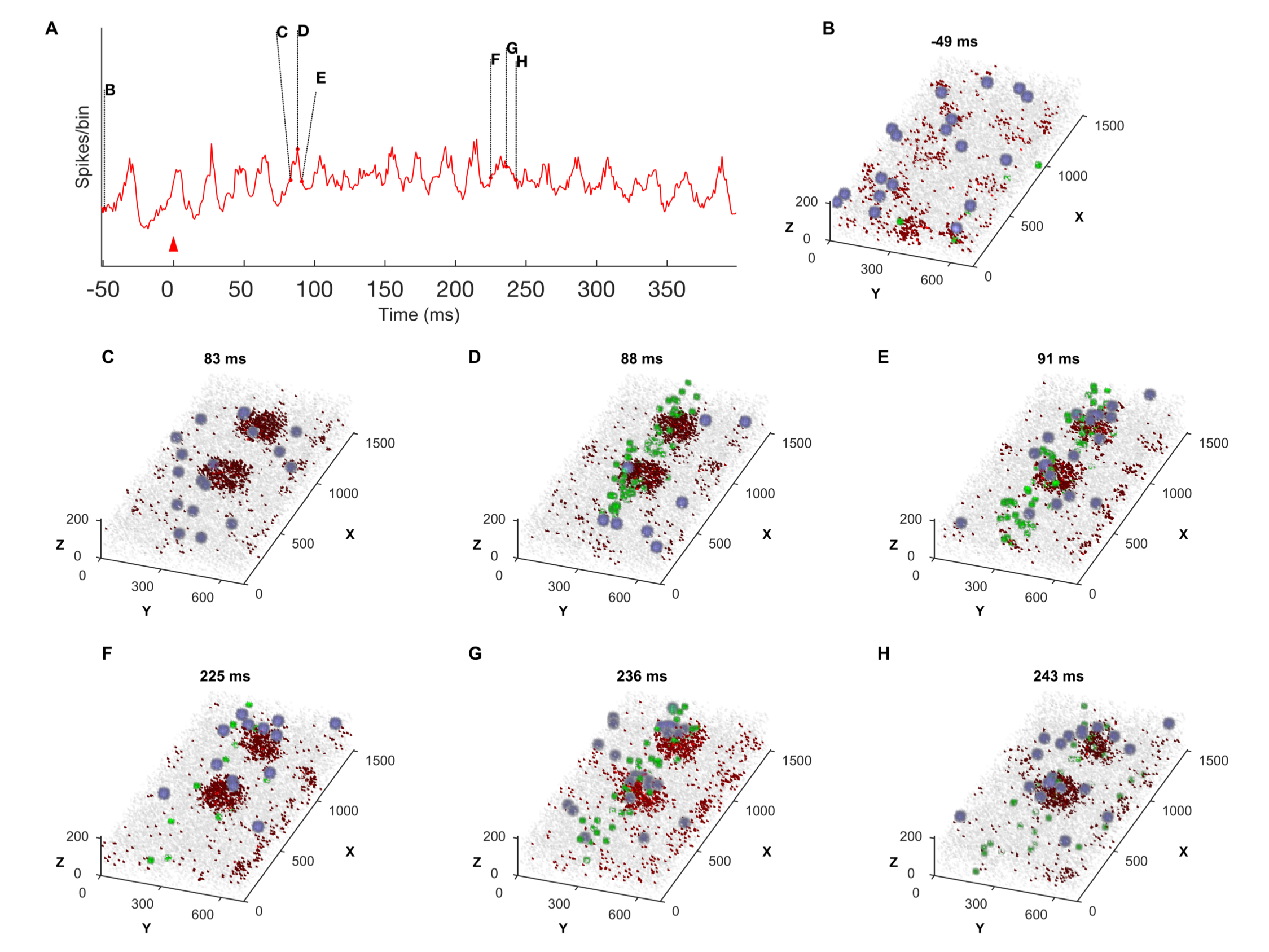

Supplement: S3 Fig — A: GrC population PSTH showing the timing of volumetric maps at various points during the network activity. B-G: Volumetric maps that portray the network activity at various time points. The stimulus onset is at 0 ms. One can see the patchy mossy fiber activation, GrC spiking activity in response to that and GoC activity all along the transverse axis. The colors represent the same scheme as explained in S1 Movie. (TIFF) [file pcbi.1005754.s004.tiff]

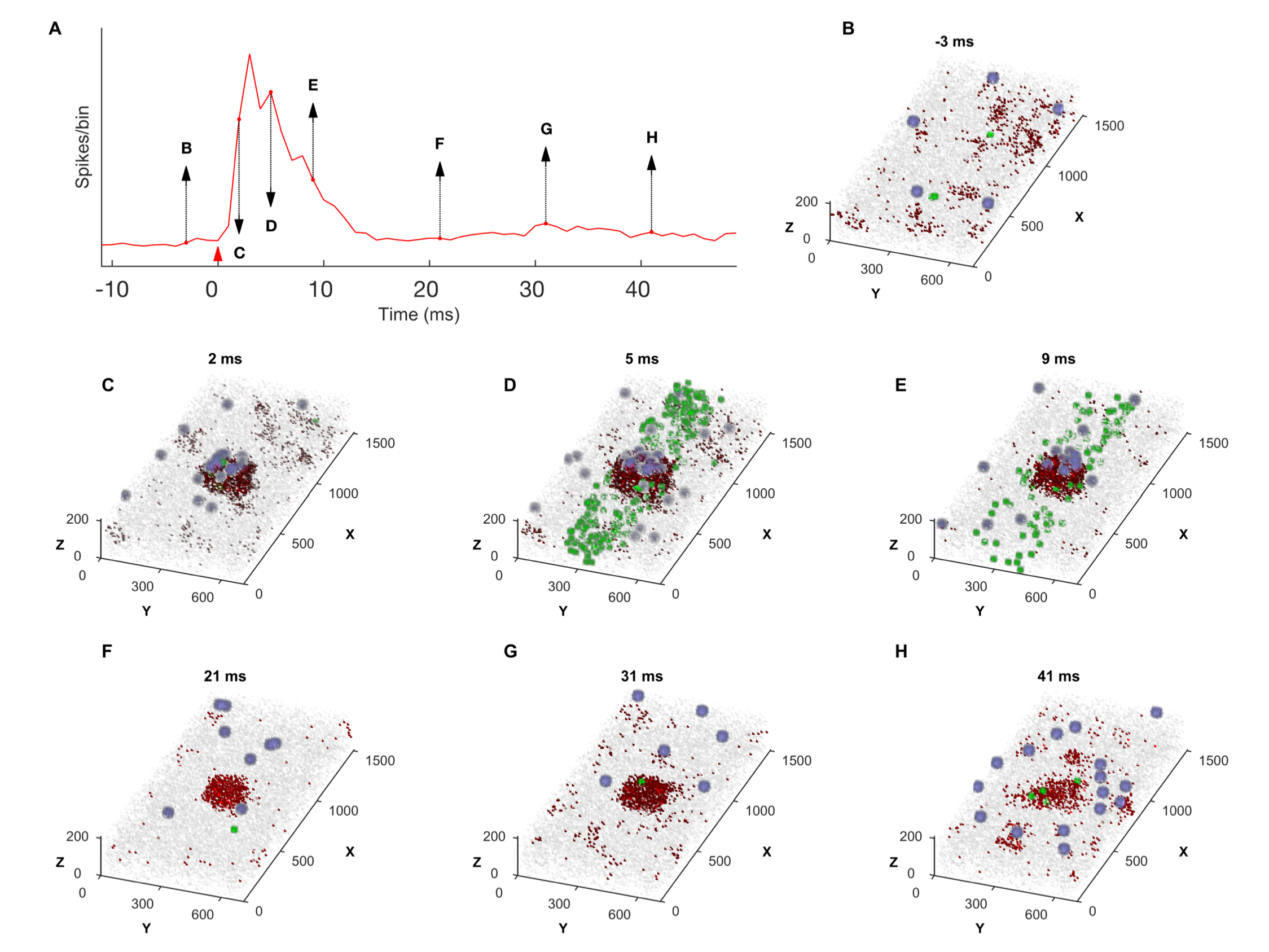

Supplement: S4 Fig — A: GrC population PSTH showing the timing of volumetric maps at various points during the network activity. B-G: Volumetric maps that portray the network activity at various time points. The stimulus onset is at 0 ms. One can see the mossy fiber burst, GrC bursting activation, GoC activity all along the transverse axis. Network rebound response can also be seen from panel G. The colors represent the same scheme as explained in S1 Movie. (TIFF) [file pcbi.1005754.s005.tiff]

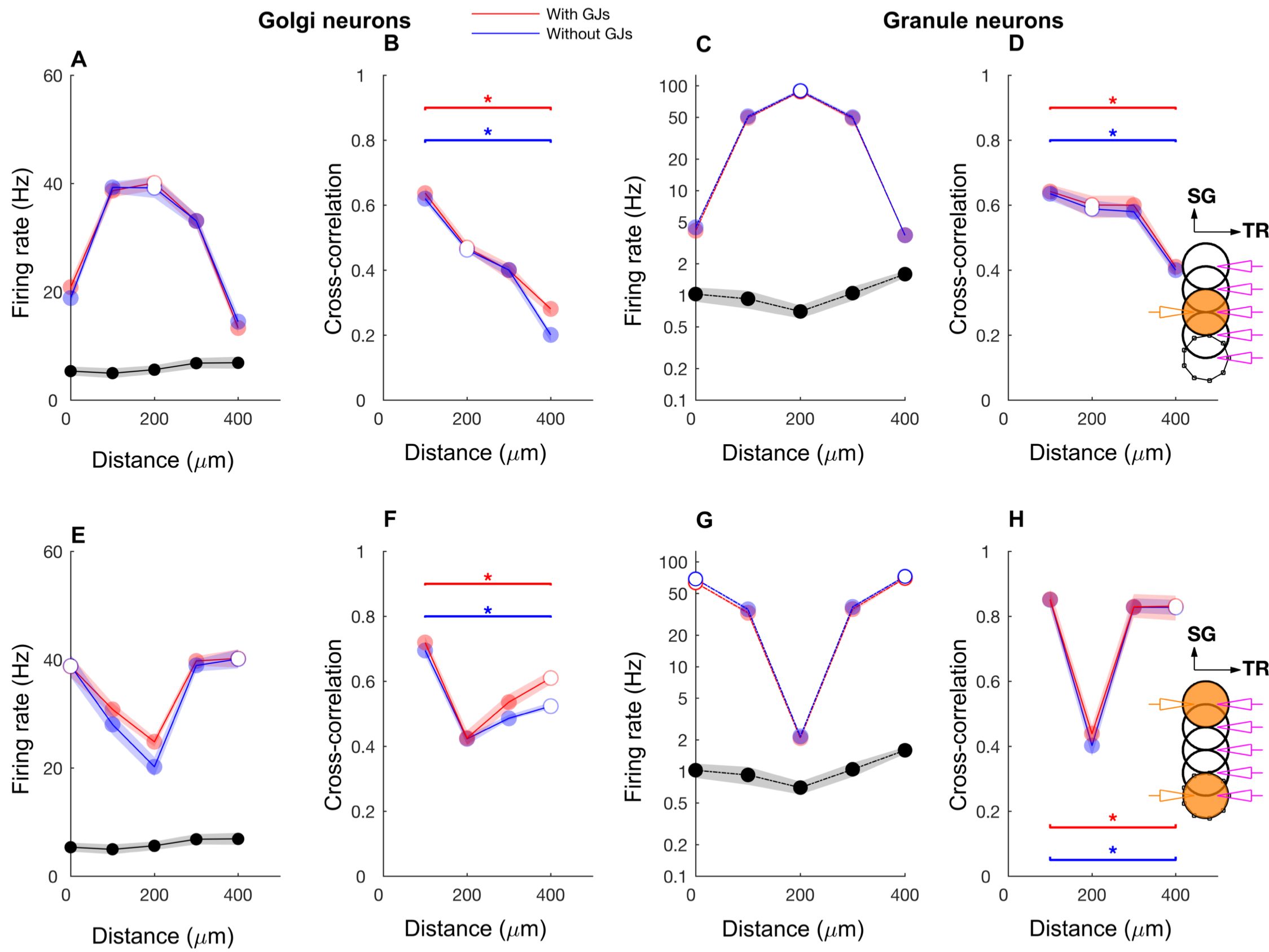

Supplement: S5 Fig — A-D: GoC firing rate (A), cross-correlation (B), GrC firing rate (C), cross-correlation (D) along the sagittal axis when the network was activated with a single patch of mossy fibers with bursting inputs. Black lines represent background network firing rate for the respective patches. E-H: Same as A-D with two ON patches separated by 400 μm along the sagittal axis with bursting mossy fibers inputs. Asterisk and triangle represent significant (p<0.01) and insignificant correlation (p> = 0.01), respectively. The stimulation and recording configuration shown as insets in D and H represent the same configurations as those in Fig 6. (TIFF) [file pcbi.1005754.s006.tiff]

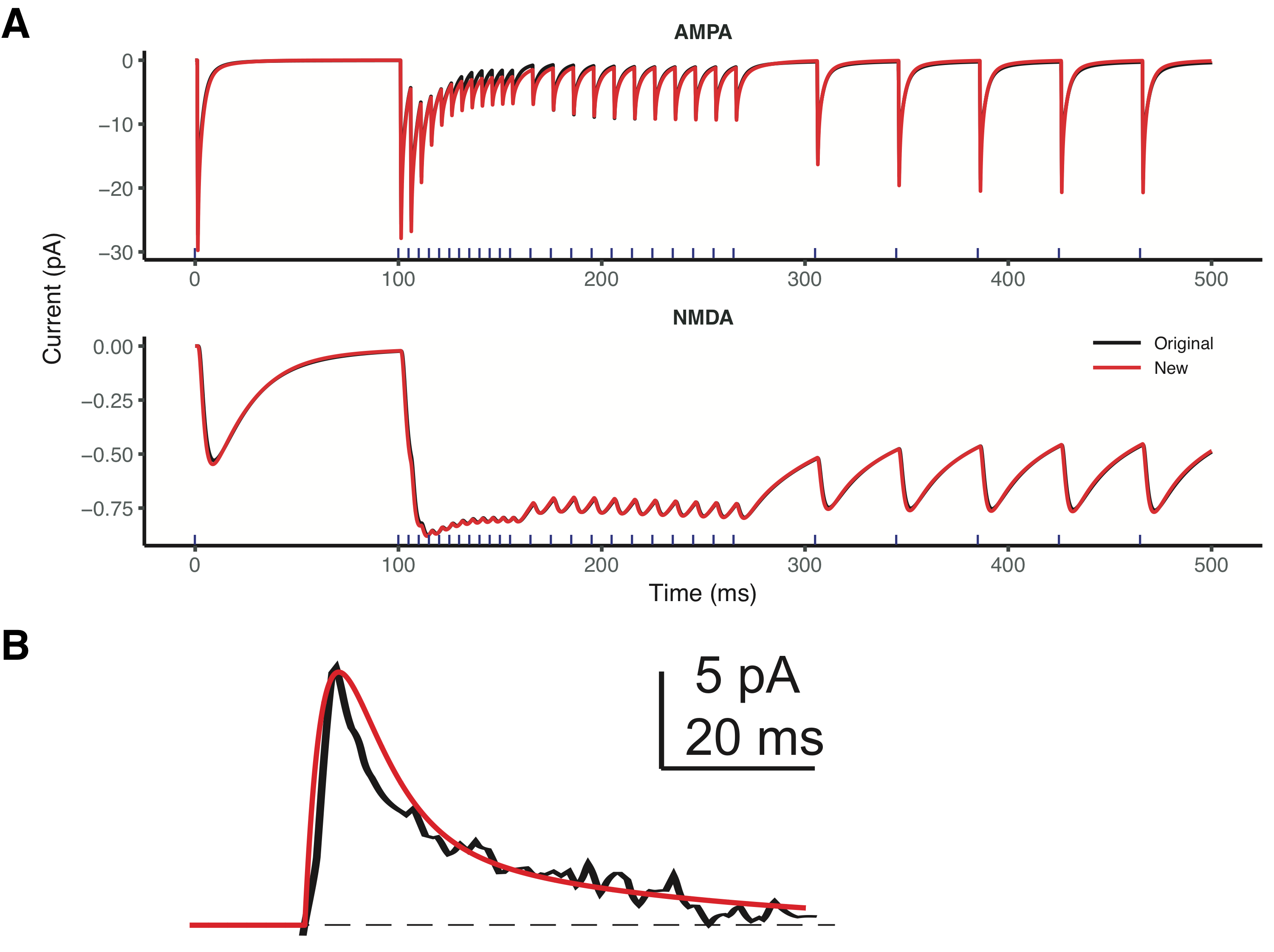

Supplement: S6 Fig — A: Black lines are the AMPA and NMDA current induced by presynaptic spikes (blue) in the original model [79]. Red lines are the same currents in our model where the glutamate diffusion is approximated by a cascade linear process (Eq 2). B: An experimental data of GrC eIPSC copied from [13] (black), and eIPSC of our GABAergic synapse model in the same condition (red). (TIFF) [file pcbi.1005754.s007.tiff]
